# Operando Luminescence Thermometry for Hydrocarbon Conversion Catalysis: Dealing with Dynamic Changes in Catalyst Optical Properties

**DOI:** 10.1021/acsami.5c00243

**Published:** 2025-04-01

**Authors:** Robin Vogel, Daniël W. Groefsema, Maria A. van den Bulk, Thimo S. Jacobs, P. Tim Prins, Freddy T. Rabouw, Bert M. Weckhuysen

**Affiliations:** † Inorganic Chemistry and Catalysis Group, Institute for Sustainable and Circular Chemistry, Utrecht University, Universiteitsweg 99, Utrecht 3584 CG, The Netherlands; ‡ Soft Condensed Matter Group, Debye Institute for Nanomaterials Science, Utrecht University, Princetonplein 1, Utrecht 3584 CC, The Netherlands

**Keywords:** thermometry, temperature sensing, luminescence, phosphor material, fluorescence, propane dehydro

## Abstract

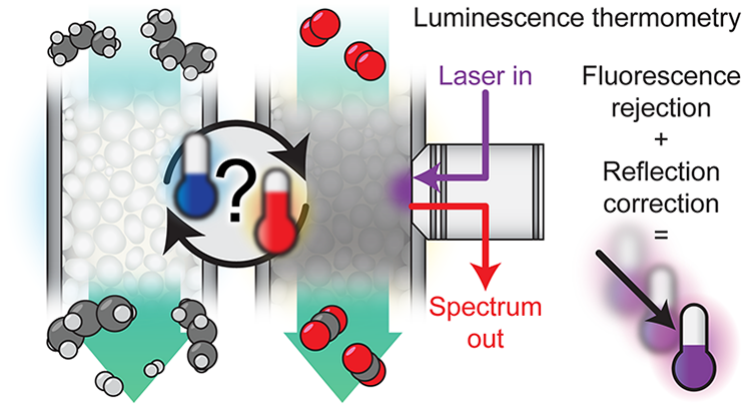

Luminescence thermometry offers an attractive strategy for temperature sensing of a catalytic process, whereby the temperature-dependent luminescence of a phosphor material embedded in the reactor is excited and recorded remotely. However, changes in the optical properties of the catalyst materials can distort the luminescence recording, resulting in inaccurate temperature sensing. This is particularly problematic in hydrocarbon conversion catalysis due to the buildup of carbon deposits, which color the material and cause fluorescence upon excitation. In this work, we developed operando reflectance-corrected time-gated luminescence thermometry, using a highly dynamic and industrially relevant Pt–Sn-based propane dehydrogenation (PDH) catalyst and an Eu^3+^-based phosphor as a showcase. PDH catalyst materials deactivate via the deposition of carbon, which is subsequently removed by an oxidative regeneration treatment. During these alternating reaction–regeneration processes, the background fluorescence and the color of the catalyst material change continuously. This skews the Eu^3+^ luminescence, leading to temperature-readout artifacts. We solved these problems by rejecting background fluorescence using time-gated detection following pulsed excitation and by correcting for the wavelength-dependent absorption changes of the PDH catalyst. This method offers accurate temperature sensing of the PDH catalyst material and is a step forward in the development of luminescence thermometry for samples with highly dynamic optical properties.

## Introduction

The operation temperature of catalyst materials that drive chemical conversion reactions has a key influence on the efficiency, stability, and safety of the process. In this regard, direct and accurate temperature sensing of catalyst materials at work offers real-time control. Moreover, the temperature influences the thermodynamics and the kinetics of a chemical conversion process. The catalytic dehydrogenation of light alkanes—an industrial route toward important commodity chemicals, such as propylene—can be accelerated by increasing the temperature of the catalyst bed. Dehydrogenation catalyst materials typically suffer from short-term deactivation due to the buildup of carbon deposits (coke) on the surface of the catalyst. The activity is recovered by coke combustion with an oxidative-regeneration step. High temperatures during the regeneration trigger the sintering of the active phase, resulting in activity loss.^1−6^ Temperature monitoring of the catalyst bed can offer more control over the reaction conditions, allowing for enhanced stability and activity of the catalyst material.

Luminescence thermometry offers a strategy for remote temperature sensing. This involves the use of phosphors—in close contact with the catalyst—that are excited with a light source and exhibit temperature-dependent luminescence.^7,8^ A wide variety of luminescence features can be used to deduce the temperature of the phosphor, such as luminescence lifetime,^9^ intensity, color (peak position), and the ratio between emission lines of two emitters with different thermal quenching mechanisms.^10−12^ Boltzmann thermometry is a particularly popular strategy: recording the luminescence intensity ratio (LIR) of two thermally coupled emissive states that follow Boltzmann statistics.^13,14^ This method is accurate and precise and is not cross-sensitive to variations in absolute luminescence intensities. Luminescence thermometry is relatively noninvasive compared to the insertion of a thermocouple in the catalyst bed, which could influence the flow of reactants and heat through the catalyst bed or affect the chemical-reaction pathways.^15^ In addition, luminescence thermometry based on visible light offers a higher spatial resolution than infrared thermography.^16,17^ Other promising thermometry methods for catalysis research are based on NMR spectroscopy and X-ray absorption spectroscopy.^18,19^

Despite the advantages of Boltzmann thermometry, previous experiments have shown that the method is sensitive to a variety of potential artifacts. Spectral distortions due to effects other than temperature variations can result in erroneous temperature readouts. The measured luminescence can be modulated by parameters such as wavelength-dependent transmission, absorption, and scattering by the sample or the thermometer material and background fluorescence.^12,17,20,21^ These artifacts can be mitigated with a careful calibration of the phosphor in its measurement environment but only if these parameters do not change during the thermometry experiment.^17,21^ Errors in temperature readout due to spectral distortions might be prevented with luminescence lifetime thermometry. However, lifetime thermometry comes with its own cross-sensitivities to parameters that change during catalysis, such as the refractive index of the sample, the presence of Förster resonance energy transfer (FRET) acceptors, or the O_2_ concentration in the gas feed.^15,22,23^

Luminescence thermometry has been successfully applied to measure the temperature of catalysts at work.^17,24−29^ Reported thermometry experiments highlighted temperature increases in catalyst materials during exothermic hydrocarbon conversion processes.^26,27^ Most of these applications involve static catalyst materials that do not suffer from coking and consequent color changes during catalysis, except for the work of Geitenbeek et al.^27^ The highly dynamic nature of a coking catalyst might compromise luminescence thermometry. In the case of a coking catalyst, the excitation source that is used to induce phosphor luminescence can also induce background fluorescence due to electronic transitions of polyaromatic coke species that form on its surface.^30,31^ The constant evolution of the hydrocarbon species on the surface of a coking catalyst causes ever-changing background fluorescence, instantly outdating any calibration data.^30−32^ Moreover, wavelength-dependent absorption changes can skew the luminescence spectrum recorded during the coking of a catalyst, yielding major temperature readout artifacts.^12,33,34^ This potential source for biased sensing can again be mitigated with a local calibration,^17,21^ but this will be outdated as soon as the appearance of the catalyst changes. Indeed, numerous operando UV–vis experiments have shown that the absorption characteristics of catalysts change continuously, due to the progressive blackening during coking.^35,36^ These issues have not yet been addressed for the luminescence thermometry of catalysts.

In this work, we develop operando Boltzmann luminescence thermometry for highly dynamic coking propane dehydrogenation (PDH) catalyst materials, tackling both background fluorescence and wavelength-dependent absorption artifacts. We first show that temperature measurements with a thermally stable Eu^3+^-based Boltzmann thermometer and conventional time-integrated detection are inaccurate because of background fluorescence. Time-gated thermometry with pulsed excitation, followed by delayed—time-gated—detection, allows the fluorescence to decay before recording the slower decaying Eu^3+^ luminescence that carries the temperature information, yielding background-fluorescence-free Eu^3+^ luminescence spectra.^37−40^ We optimize this measurement strategy with a theoretical model using the time dynamics of Eu^3+^ luminescence and background fluorescence upon pulsed excitation.^41^ Time gating improves the accuracy of the operando luminescence thermometry measurements, but the recorded temperature is also skewed by wavelength-dependent absorption changes due to progressive blackening of the catalyst. Quickly alternating—on a time scale of seconds—between reflectance spectroscopy and time-gated thermometry modes offers a strategy to correct for the continuously evolving appearance of the coking catalyst material.^42−44^

## Results and Discussion

**Figure 1:**
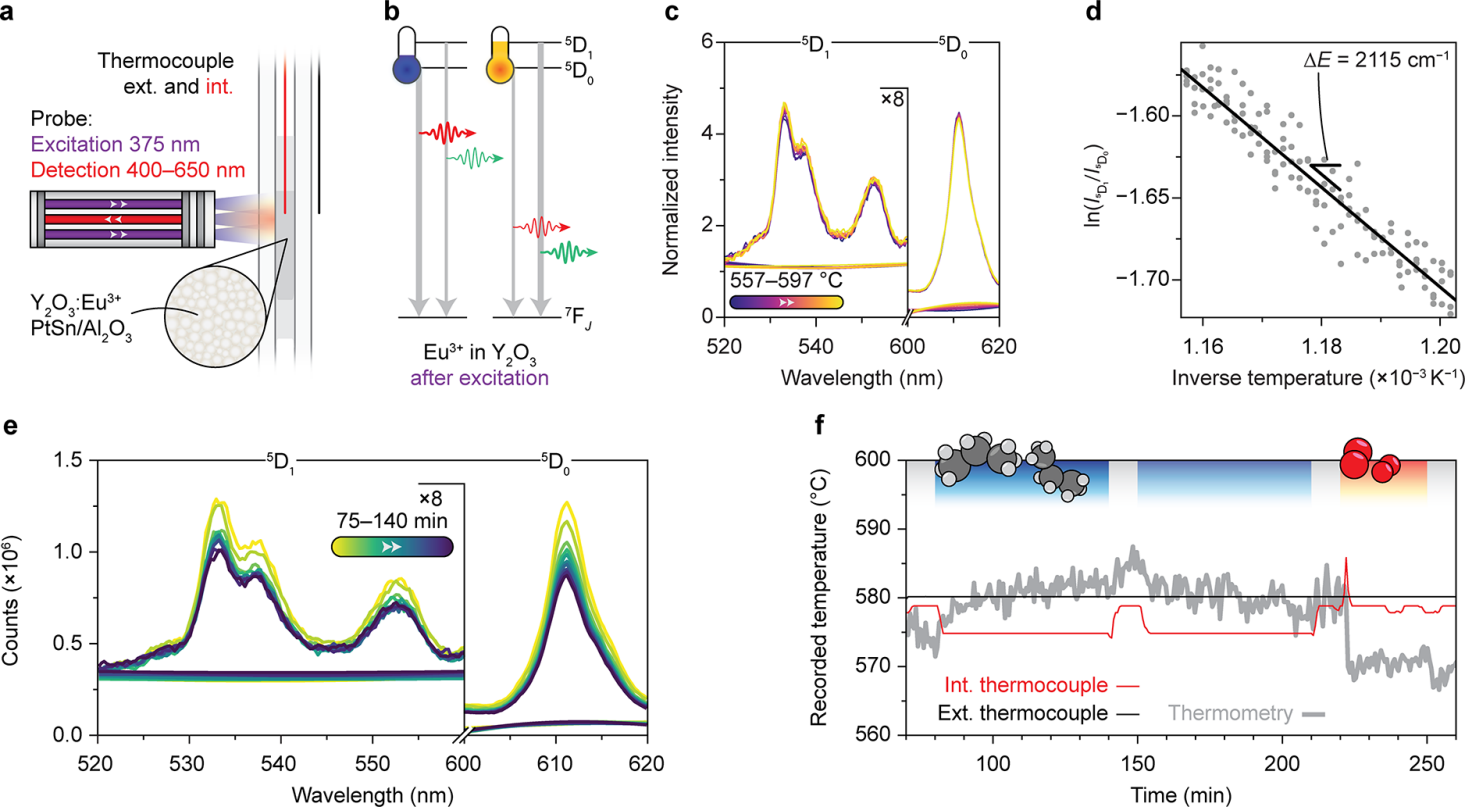
Operando luminescence thermometry for propane dehydrogenation (PDH) catalysis. (a) Schematic overview of the experimental setup for luminescence thermometry with the catalyst and temperature-sensor material PtSn/Al_2_O_3_–Y_2_O_3_/Eu^3+^ (150–425 μm) (Figure S1). The online gas chromatography (GC) results are discussed in Figure S2. (b) Simplified energy-level diagram of Eu^3+^ in Y_2_O_3_. The temperature-sensing method is based on the ratio of the intensities of the emission from the thermally coupled ^5^D_1_ and ^5^D_0_ energy levels. (c) Normalized Eu^3+^ luminescence spectra recorded during the calibration. The spectra are shown together with their backgrounds obtained from a spectral fit (Figure S3b). The spectra are normalized such that their integrated areas equal 1. (d) Calibration of the thermometer with the background-corrected intensities attributed to ^5^D_0_ and ^5^D_1_ emission. The inverse temperature (557–597 °C) is plotted against the natural logarithm of the ratio of ^5^D_1_ to ^5^D_0_ emission (gray), along with a fitted line (black). The slope of this line is proportional to a Δ*E* of 2115 cm^–1^. (e) Eu^3+^-luminescence spectra recorded during PDH. The spectra are shown together with their backgrounds obtained from a spectral fit (Figure S3b). (f) Recorded temperatures during the experiment using an external thermocouple (black), an internal thermocouple (red), and luminescence thermometry (gray). For each spectrum, the LIR of the background-corrected ^5^D_0_ and ^5^D_1_ emission (522–559 and 605–621 nm, respectively) is converted to a temperature value using the calibration data (panel d). All temperature traces are obtained by averaging the temperature data every 40 s.

We set out to apply Boltzmann luminescence thermometry to monitor the temperature of an industrially relevant propane dehydrogenation catalyst material under working conditions. The results of the initial attempt are illustrated in Figure 1. The experimental setup is summarized in Figure 1a. In short, the catalyst (PtSn/Al_2_O_3_) and temperature-sensor material (Y_2_O_3_/Eu^3+^) were mixed and placed in a quartz reactor tube in an oven. The Eu^3+^-based phosphor was excited with a 375 nm laser using a fiber-coupled optical probe, creating an excitation spot of approximately 5 mm in diameter on the powder mixture. The resulting luminescence was collected with the same probe and analyzed with a spectrometer. The temperature inside the oven was monitored and controlled with an external thermocouple outside the reactor (black in Figure 1a; see also Figure S1). The internal thermocouple (red in Figure 1a) was used to probe the temperature in the reactor bed for validation of the luminescence thermometry results, although it is an invasive element in the reactor bed that can distort the catalysis. The experiment involved three stages: (1) the luminescent thermometer sensor material was calibrated with a temperature sweep under He flow, after which (2) the oven temperature was maintained constant while the endothermic propane dehydrogenation (PDH) reaction started. Over time, coke formed on the surface of the catalyst material, hampering the propane conversion process. (3) In the final step, the coke was combusted using oxygen to regenerate the catalyst. The catalytic activity was monitored with online gas chromatography (GC). The conversion of propane decreased from 50% to 40% over the course of the PDH phase—lasting 2 h—as coke gradually blocked the active sites of the catalyst material. The selectivity toward the desired product, propylene, was approximately 97% (Figure S2).

The working principle and the calibration of our luminescence thermometer are discussed in Figure 1b,c. The energy-level scheme of Eu^3+^ in Y_2_O_3_ (Figure 1b) illustrates the thermometry approach in this work. After excitation, the population of the thermally coupled ^5^D_0_ and ^5^D_1_ energy levels follows Boltzmann statistics in the temperature range used for the reported experiments.^13^ As a result, the ratio of the emission intensities from the ^5^D_1_ to ^5^D_0_ levels increases with the rising temperature. Indeed, the normalized spectra recorded during the calibration show that the bands due to ^5^D_1_ emission become more intense relative to the bands due to ^5^D_0_ emission (Figure 1c). Besides the well-defined Eu^3+^ luminescence features, we also observed spectrally broad background fluorescence with a constant intensity, probably due to the excitation of a small amount of organic impurities in our sample. The natural logarithm of the background-corrected luminescence intensity ratio (LIR) of the ^5^D_1_ to ^5^D_0_ emission scales linearly with the inverse temperature. The slope of the line yields a fitted value of Δ*E* = 2115 cm^–1^, i.e., the apparent energy difference between the ^5^D_0_ and ^5^D_1_ energy levels (Figure 1d). This deviates significantly from the literature value of 1740 cm^–1^, likely due to spectral distortions because of an inaccurate background correction.^45^ Nevertheless, we used these calibration data in our initial attempt to track the temperature of the catalyst bed during the PDH and regeneration phases of the experiment. Besides the spectral distortions, spectral overlap between ^5^D_0_ and ^5^D_1_ emissions can cause deviations from the Boltzmann behavior of the LIR. We estimated the effect of this spectral overlap on our thermometry experiments and found that our calibration procedure accounts for this overlap with a systematic error of 0.003 °C remaining, which is negligible compared to other experimental uncertainties (Figure S4).

The changing ratio between background fluorescence and Eu^3+^ luminescence during PDH (Figure 1e) complicates accurate temperature sensing. The gradual buildup of coke reduces the absolute luminescence intensity over the course of the reaction as a fraction of both the excitation and emission light is absorbed increasingly.^4,6^ In the meantime, the background fluorescence intensity increases slightly. As a result, the total signal in the ^5^D_1_ spectral region—due to a combination of Eu^3+^ luminescence and background fluorescence—increases relative to that in the ^5^D_0_ spectral region. Without background correction, this leads to an apparent temperature increase of 250 °C during the endothermic PDH reaction (Figure S3a). This underlines the necessity of a correction for the background fluorescence.

We attempted a background fitting procedure (Figure S3b), but we are unable to obtain a LIR-based temperature in agreement with the temperature recorded by the internal thermocouple during PDH and oxidative regeneration (Figure 1f). Upon introduction of propane at 80 min (after calibration), the endothermic PDH reaction starts, and the heat required for the formation of propylene is withdrawn from the catalyst bed. At this point, the internal thermocouple senses a temperature decrease of 4 °C, while the external thermocouple maintains the temperature outside the reactor constant at 580 °C. When switching back to a He flow (140 and 150 min and 210–220 min), the endothermic reaction stops. Indeed, during these inert periods, the internal thermocouple registers a temperature 4 °C higher than that during reaction periods. Our luminescence thermometry sensor does not register the temperature variations caused by the endothermicity of the PDH reaction. Instead, the temperature appears to increase gradually between 80 and 150 min. The combustion of carbon deposits begins after 210 min when oxygen is introduced. At this point, the internal thermocouple registers a temperature spike of 8 °C for about 2 min. This heating effect is also not detected with a luminescence thermometer. Clearly, our simple background-subtraction procedure for luminescence spectra yields insufficient temperature accuracy.

### Time-Gated Luminescence Thermometry

We can prevent background fluorescence from reaching the detector completely with the aid of time gating for more accurate luminescence thermometry. Fortunately, background fluorescence of organic molecules has a short lifetime on the order of nanoseconds compared to lanthanide luminescence on the order of milliseconds. We used pulsed excitation, followed by delayed—time-gated—detection to reject background fluorescence and obtain clean Eu^3+^ luminescence spectra, using an intensified scientific complementary metal-oxide semiconductor (sCMOS) camera.

**Figure 2:**
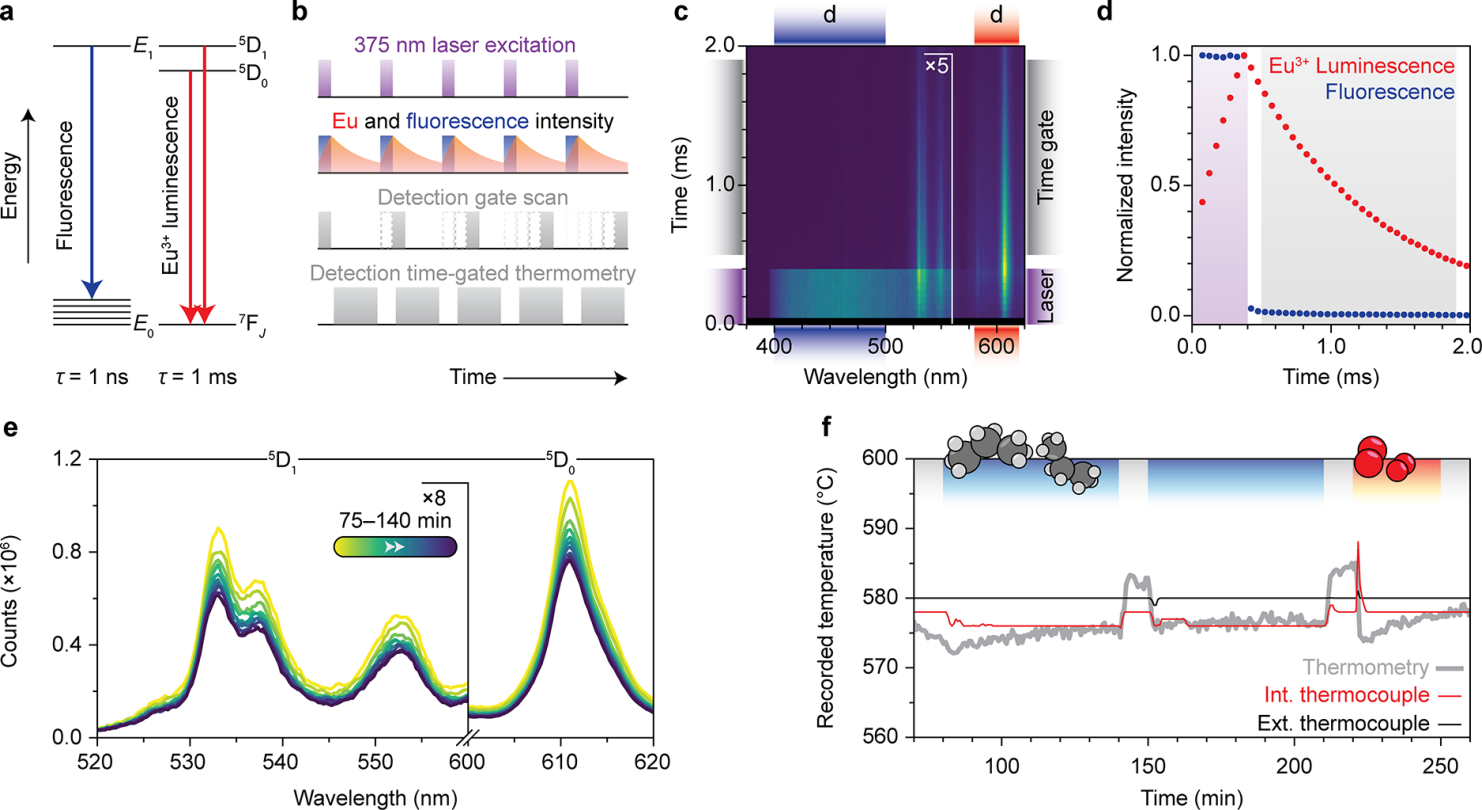
Operando time-gated luminescence thermometry for propane dehydrogenation (PDH) catalysis. (a) Schematic representation of fluorescence and Eu^3+^ luminescence. (b) Measurement schemes for time-resolved spectroscopy (detection gate scan, panels c and d) and time-gated spectroscopy (panel e). The detection gate scan illustration is not to scale for the shown measurements. (c) Heatmap of time-resolved spectroscopy of the catalyst and temperature-sensor material PtSn/Al_2_O_3_–Y_2_O_3_:Eu^3+^ (150–425 μm) at 580 °C. The signal resulting from excitation with a 375 nm block-pulse (2 ms period, 20% duty cycle) is recorded by scanning the center of the time gate (50 μs width) in 39 increments of 50 μs from 75 to 1975 μs. (d) Normalized time traces of background fluorescence (400–500 nm) and Eu^3+^ luminescence (580–620 nm). The laser pulse is illustrated with the purple rectangle; the time gate used in panel e is illustrated with the gray rectangle. (e) Time-gated Eu^3+^-luminescence spectra recorded during PDH. The spectra are recorded using a 375 nm block-pulse (2 ms period, 20% duty cycle) and time-gated detection between 0.5 and 1.9 ms. (f) Recorded temperature during the experiment, using an external thermocouple (black), an internal thermocouple (red), and time-gated luminescence thermometry (gray). All temperature traces are obtained by averaging the temperature data every 40 s. The luminescence thermometry temperature is calculated using the time-gated luminescence intensity ratio of ^5^D_1_ to ^5^D_0_ emission (522–559 and 605–621 nm, respectively) and a calibration, analogous to Figure 1, that yielded Δ*E* = 1700 cm^–1^.

Figure 2 illustrates the development of operando time-gated luminescence thermometry for the PDH process. The sample and procedure are the same as those in Figure 1 but now with time-gated detection. Figure 2a shows that laser excitation induces the fluorescence of organic molecules on the surface of the sample and luminescence of the Eu^3+^-based phosphor. Eu^3+^ luminescence involves forbidden *f*–*f* transitions, the lifetime of which is typically on the order of milliseconds,^9^ many orders of magnitude slower than the nanosecond-time scale fluorescence of organic molecules.^37^ The dynamics of both processes were studied with time-resolved spectroscopy to find optimal settings for time-gated thermometry (Figure 2b, gate scan).

The time-resolved spectra (Figure 2c,d) reveal the strongly different time dynamics of organic fluorescence and Eu^3+^ luminescence upon block-pulse excitation. The excitation, during the on-period of the laser, results in a spectrally broad (400–500 nm) instantaneous rise in signal intensity due to fluorescence. In addition, the signal intensity of the well-defined Eu^3+^-luminescence lines (520 and 630 nm) rises more slowly. The fluorescence disappears within 50 μs after the laser is switched off, in line with the expected nanosecond fluorescence lifetime. In contrast, the Eu^3+^ luminescence decays exponentially over a time scale of milliseconds.

We optimized time gating for background fluorescence rejection based on the time dynamics of the background fluorescence and Eu^3+^ luminescence. Operando time-gated luminescence thermometry is more precise with optimization of the fluorescence-free Eu^3+^ count rate, aimed at optimizing the signal-to-noise ratio (SNR) of the recorded Eu^3+^ luminescence spectra.^32^Figure 2d shows that background fluorescence is absent between 100 μs after a laser pulse until 100 μs before the next laser pulse. This leaves the pulse frequency (or period length) and duty cycle of the laser as parameters that influence the time-gated luminescence count rate. We formulated a model to describe the rise and decay dynamics of Eu^3+^ luminescence using block pulse excitation,^41^ considering ingrowth of population due to incomplete depletion of the Eu^3+^ luminescence at the end of the period (Figure S5). We used the rise and decay rates of Eu^3+^ luminescence that our sample exhibited and found that the 2 ms period and 20% duty cycle yield a close-to-optimal count rate (Figure S6).

Optimized operando time-gated thermometry yields background-fluorescence-free Eu^3+^ luminescence spectra. We used 375 nm block-pulse laser excitation with a 2 ms period and 20% duty cycle and recorded the fluorescence-free part of the signal pulse between 0.5 and 1.9 ms of the 2 ms period (Figure 2b, time-gated thermometry). Comparing the time-integrated spectra during PDH from Figure 1e to the time-gated spectra from Figure 2e shows that time-gated luminescence thermometry effectively rejects background fluorescence.

The time-gated LIR-based temperature is in closer agreement with the temperature recorded by the internal thermocouple during the catalysis and oxidative regeneration steps, as shown in Figure 2f. It is encouraging to see that the temperature drops when the reactant gas propane is introduced, at *t* = 150 min, and that the temperature increases when switching back to He, at *t* = 210 min, in accordance with the endothermicity of the PDH process. The LIR-temperature does not register all of the temperature jumps that are recorded by the internal thermocouple. The inability to detect the expected endothermic and exothermic effects during the different stages of the experiment is evident, especially at the beginning of the PDH and oxidative regeneration phases (*t* = 80 and 220 min, respectively), where the color of the sample changes rapidly due to the formation and combustion of coke. Furthermore, the LIR-temperature starts to deviate more and more over the course of the PDH reaction phase (80–210 min) as the sample darkens gradually due to coke build-up.

### Reflectance-Corrected Time-Gated Luminescence Thermometry

We attribute the inaccuracy of the LIR-based temperature sensing, as presented in Figure 2f, to the changing color of the catalyst material during the PDH reaction and oxidative regeneration due to the formation and combustion of coke. The evolution of the carbon deposits during these processes causes their optical properties to change continuously.^35,36^ These wavelength-dependent absorption and scattering changes skew the shape of Eu^3+^ luminescence spectra and influence the LIR, making accurate temperature sensing of this dynamic sample challenging.

**Figure 3:**
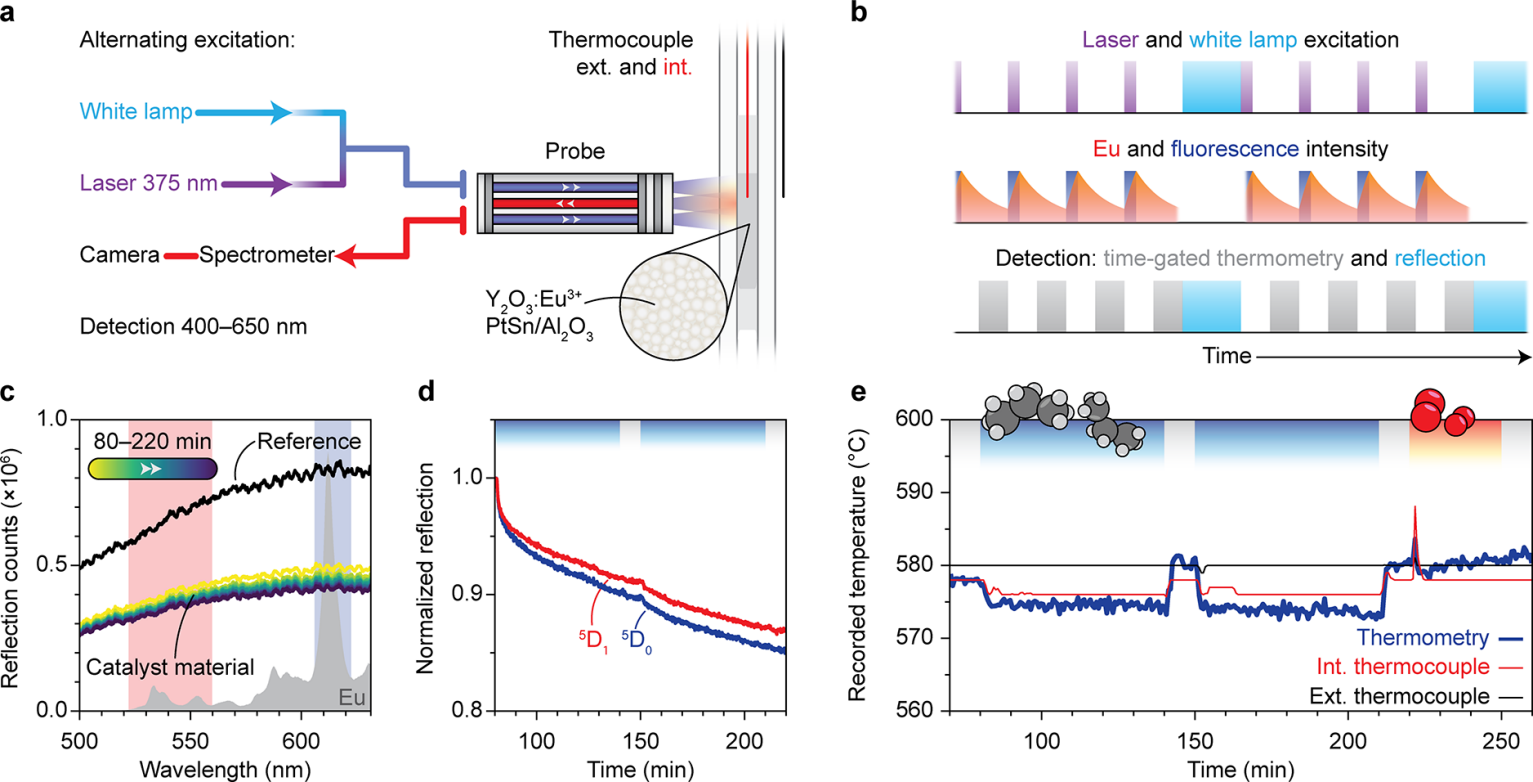
Operando reflectance-corrected time-gated luminescence thermometry for propane dehydrogenation (PDH) catalysis. This figure uses the same data as Figure 2 but now includes the reflectance correction. (a) Simplified and schematic experimental setup and (b) timing diagram for the reflectance-corrected time-gated operando luminescence thermometry method (Figure S1). The timing diagram illustration is not to scale for the measurement presented. (c) Reflection spectra recorded during PDH. A typical time-gated Eu^3+^-luminescence spectrum is presented in gray. The spectral regions in which ^5^D_1_ and ^5^D_0_ emission occurs, used for our thermometry calculations, are marked in red and blue, respectively. The reflection spectra of the catalyst and temperature-sensor material are colored; the spectrum of the white reference material, polytetrafluoroethylene (PTFE) powder, is depicted in black. (d) Averaged and normalized reflection intensity time traces of the spectral regions where ^5^D_1_ (522–559 nm) and ^5^D_0_ (605–621 nm) emission from Eu^3+^ occurs. (e) Recorded temperature during the experiment, using an external thermocouple (black), an internal thermocouple (red), and reflectance-corrected luminescence thermometry (blue). All temperature traces are obtained by averaging the temperature data every 40 s. The luminescence thermometry temperature is calculated using the luminescence intensity ratio of ^5^D_1_ to ^5^D_0_ emission (522–559 and 605–621 nm, respectively) and a calibration analogous to Figure 1 that yielded a Δ*E* of 1776 cm^–1^.

We adjusted our experimental approach to be able to track and compensate for the changing color of the catalyst and temperature-sensor particles using reflectance spectroscopy data (Figure 3). Specifically, we used a bifurcated fiber to couple both the 375 nm laser light and a white lamp in the fiber-coupled optical probe (Figure 3a). The laser modulation and lamp shutter were synchronized to the accumulation periods on the spectrometer. In this way, we alternated between time-gated luminescence thermometry and reflection spectroscopy modes, yielding time-gated Eu^3+^ luminescence and reflection spectra (Figure 3b).

Figure 3c shows the time-dependent reflection spectra of the powder mixture of catalyst and temperature-sensor particles. Clearly, the reflectance decreases during the PDH reaction step of the experiment due to coking. This is consistent with our earlier observation that coking led to a decrease in Eu^3+^ luminescence intensity over the course of the PDH stage, due to increasing absorption of the excitation and emission light (Figure 2e). The buildup of carbon deposits is also shown in the online GC data. Coking results in a gradual decrease of propane conversion (Figure S2). In fact, the decrease in propane conversion correlates strongly with the darkening of the sample; therefore, the reflection measurements can be used to monitor the performance of the catalyst material directly (Figure S8).

Importantly, we now observe that the reflectance changes to a different extent in the two wavelength regions of interest (Figure 3d). Over the course of 2 h, the reflectance decreases to 87% of the initial value in the spectral region of the ^5^D_1_ emission lines and to as low as 85% in the ^5^D_0_ spectral region. For accurate temperature sensing, the shapes of the Eu^3+^ luminescence spectra should be corrected for these wavelength-dependent changes in the absorption.

Each Eu^3+^ luminescence spectrum is corrected for wavelength-dependent sample absorption and scattering with the two reflection spectra that were recorded before and after its acquisition (Figures S9–S11).^42,43^ The procedure is an adaptation to the correction method for Raman scattering intensities proposed in ref (43). The adaptations necessary to account for the differences in reflectance between the excitation wavelength and the multiple emission wavelengths are detailed in Figures S9 and S10. We find that the recorded Eu^3+^ luminescence spectrum ψ(λ) must be converted into a corrected spectrum
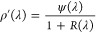
1where *R*(λ) is the wavelength-dependent reflectance of the sample. As our experiment alternates between measurements of ψ(λ) and *R*(λ), our procedure accounts for dynamic changes in sample absorption and scattering.

A complicating factor in our procedure is the sensitivity of the recorded reflection spectrum to setup alignment. To calculate the sample reflectance *R*(λ), we must account for the lamp spectrum as well as for light reflection on the quartz windows and internally in the metal-clad fiber probe (Figure S9; eq S16). However, reference measurements of a white polytetrafluoroethylene (PTFE) powder in the quartz reactor reveal that the recorded light reflection depends on the placement of the fiber probe relative to the reactor (Figure S10). To account for this uncertainty in spectral shape and intensity, we include two fit parameters *x*_0_ and *x*_1_ in our calculation of the sample reflectance (eq S16), which account for the fraction of light reflected off the reactor or in the fiber probe in the spectral regions of the ^5^D_0_ and ^5^D_1_ emission, respectively. The values of *x*_0_ and *x*_1_ are optimized with a fit procedure by minimizing the temperature differences between the inert periods and the period after the regeneration (70–80 min, 142–150 min, 213.5–220.5 min, and 228–238 min, Figure 3e).

Using time gating (Figure 2) and our correction procedure (eq 1), LIR-based thermometry accurately tracks the temperature of the catalyst and temperature-sensor material during the reaction and oxidative regeneration steps (Figure 3e). The luminescence thermometry data are in close agreement with the temperature recorded by the internal thermocouple. Specifically, we observe that the temperature recorded with luminescence thermometry is more stable during the inert periods at 70–80 min, 140–150 min, and 210–220 min, compared to the uncorrected temperature (Figure 2f). During PDH, the temperature is lower by 5–10 °C, as expected from the endothermicity of PDH. Most notably, the correction procedure reveals the exothermic temperature spike at the start of the regeneration phase at 220 min, which was obscured by the rapidly changing color of the sample (Figure 2f). However, the temperature traces from luminescence thermometry and the internal thermocouple do not overlap exactly. The deviations between the magnitudes of the temperature jumps—when switching between He and reactive gases—might originate from the difference in the measurement position in the material, between the thermocouple and the luminescence thermometry. The optimization of *x*_0_ and *x*_1_ from experiment to experiment, to account for the sensitivity to setup alignment, has made the thermometry method robust and reproducible (Figure S12). The endothermic and exothermic effects of the dehydrogenation and regeneration steps are observed from measurement to measurement, albeit with differences in the magnitudes of the temperature effects, probably due to minor variations in alignment. We estimate the remaining systematic error after the correction procedure at 0.87 °C, using the variation in calculated temperatures for seven nominally identical experiments with slight differences in alignment (Figure S13) to the calibration. The precision of the method is sufficient to track the temperature of the sample. The average standard deviation of the mean measurement is 1.10 °C (Figure S13). This precision could be improved further by increasing the photon counting rates of our spectroscopy methods. Nevertheless, the method developed in this work is a step forward in the advancement of luminescence thermometry for samples with dynamic colors and fluorescent backgrounds.

## Conclusions

We developed operando luminescence thermometry for a highly dynamic and industrially important propane dehydrogenation (PDH) catalyst material by addressing the artifacts introduced by background fluorescence and wavelength-dependent absorption. The sensing method was based on the temperature-dependent luminescence intensity ratio (LIR) of the emission from two thermally coupled energy levels of a Eu^3+^-based phosphor. This phosphor was applied in a lab-scale reactor using particles, in which the catalyst and temperature-sensor materials were combined. The changing background fluorescence during the PDH process skewed the LIR, leading to enormous temperature readout artifacts. The effect of the changing background fluorescence was mitigated mathematically with a background correction to some extent, but the temperature readout was still inaccurate.

Pulsed excitation combined with delayed, time-gated detection yielded background-fluorescence-free Eu^3+^ luminescence spectra. We optimized time-gated luminescence thermometry using the time dynamics of Eu^3+^ luminescence and background fluorescence upon pulsed excitation. Time gating improved the accuracy of the temperature measurements and allowed us to detect the endothermicity of the PDH process. However, the recorded temperature was also affected by the color changes of the reactor bed during the PDH and oxidative regeneration steps.

We tracked and compensated for the wavelength-dependent absorption changes of the PDH catalyst under study with reflection spectroscopy data. Quickly alternating—on a time scale of seconds—between reflection and time-gated thermometry modes provided a data set of Eu^3+^ luminescence and reflection spectra. The operando reflection spectroscopy data correlate strongly with the performance of the catalyst material, so they can be used to probe the condition of the catalyst material directly. Moreover, the reflection spectra were used to compensate for the shape of the Eu^3+^ luminescence spectra for the changing color of the catalyst and temperature-sensor material. This operando reflectance-corrected time-gated luminescence thermometry method offered accurate temperature sensing of a highly dynamic catalyst-sensor material. This approach will be more generally applicable to other catalyst materials and spectral luminescence thermometry methods, which are all sensitive to distortions due to sample color changes and dynamic background fluorescence signals.

## Supporting Information Available

Experimental section, gas chromatography results, background correction for the time-integrated Eu^3+^ luminescence spectra, optimization of operando time-gated luminescence thermometry, analysis of the correlation between catalyst activity and its reflection, theoretical and experimental background on the reflectance correction, and analysis of the reproducibility of the reflectance-corrected time-gated luminescence thermometry method (PDF)
