# Supplementary material for: Operando Luminescence Thermometry for Hydrocarbon Conversion Catalysis: Dealing with Dynamic Changes in Catalyst Optical Properties
Source: ACS Appl Mater Interfaces. 2025 Apr 1;17(14):21215–22. doi: 10.1021/acsami.5c00243 (PMC11986894; doi:10.1021/acsami.5c00243)
Supplement: Supplementary file 1 — am5c00243_si_001.pdf [file am5c00243_si_001.pdf]

Supporting Information to

Operando Luminescence Thermometry for Hydrocarbon Conversion Catalysis: Dealing with Dynamic Changes in Catalyst Optical Properties

Robin Vogel[a], Daniël W. Groefsema[a], Maria A. van den Bulk[a], Thimo S. Jacobs[a], P. Tim Prins[a], Freddy T. Rabouw[a,b], and Bert M. Weckhuysen[a,*]

[a] Inorganic Chemistry and Catalysis Group, Institute for Sustainable and Circular Chemistry, Utrecht University, Universiteitsweg 99, 3584 CG Utrecht, The Netherlands. E-mail: b.m.weckhuysen@uu.nl

[b] Soft Condensed Matter Group, Debye Institute for Nanomaterials Science, Utrecht University, Princetonplein 1, 3584 CC Utrecht, The Netherlands

* Corresponding author. E-mail: b.m.weckhuysen@uu.nl.

Experimental Section

Chemicals and Materials

The following chemicals and materials have been used in this study: $Y_2O_3:Eu^{3+}$ (Sigma-Aldrich, 99% trace metal basis), Polytetrafluoroethylene (PTFE) powder (Sigma-Aldrich, 35 µm), carbon black powder (Cabot, VXC72R), and a 0.5 wt% Pt 1.5 wt% Sn/Al_2O_3 catalyst material (BASF, surface area of ~80 $m^2\ g^{-1}$; and pore volume of ~0.56 $cm^3\ g^{-1}$). The preparation method of the catalyst material under study is described in more detail in our earlier work.[1]

Catalyst and Temperature-Sensor Particle Preparation

The catalyst and temperature-sensor materials were combined in single particles. The Pt–Sn-based catalyst material was ground into a microcrystalline powder and mixed with an equally weighing amount of microcrystalline $Y_2O_3:Eu^{3+}$. The mixture was subsequently pressed into a pellet, crushed, and sieved to yield a 150–425 µm fraction. Within these particles, the intimate contact between the catalyst and the phosphor ensures that that the withdrawal and generation of heat due to the chemical reactions on the catalyst is sensed by the phosphor.

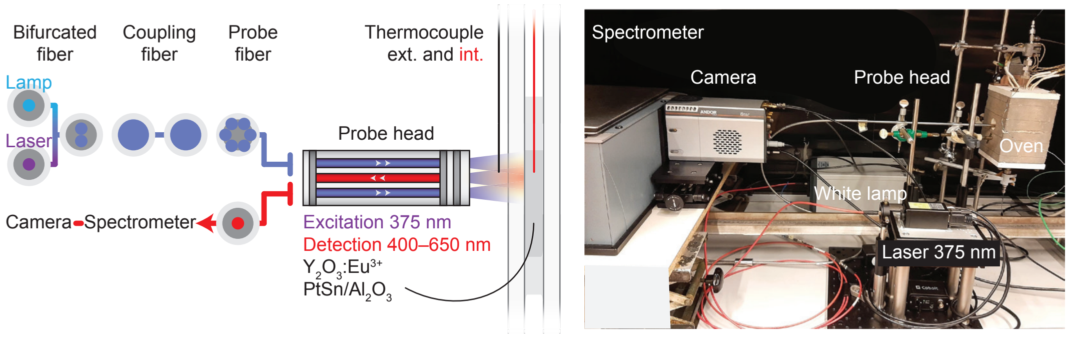

Figure S1. Experimental setup for performing reflectance-corrected time-gated *operando* luminescence thermometry of a propane dehydrogenation catalyst material. The schematic setup, together with the crosscuts of the optical fibers (purple, blue, or red colored cores), are depicted on the left. A photo of the setup is shown on the right.

Operando Luminescence Thermometry

The operando luminescence thermometry experiments were performed in a laboratory-scale reactor setup, depicted in Figure S1. A total of 240 mg of the combined catalyst and temperature-sensor material was placed in a packed-bed-type quartz reactor with flat and rectangular windows and sealed with a layer of quartz wool. The reactor was placed in an oven, with a thermocouple that was positioned in the proximity of the sample in the reactor (external thermocouple). The power output of the oven was regulated with the temperature readout of the external thermocouple, to control the temperature of the oven. The internal temperature of the top of the reactor bed was monitored with another thermocouple (internal thermocouple), as a reference for the thermometry experiments.

The catalyst was first heated from room temperature to 580 °C at a rate of 10 °C min^{-1} under a H_2 flow (8 mL min^{-1}), after which a He flow was introduced (8 mL min^{-1}) for at least 5 min. The data recording started when the calibration of the thermometer was initiated. At the same time, the reflectance-corrected and (time-gated) thermometry experiments (see below), as well as the gas chromatography (GC) program were initiated. A GC was recorded every 6 min with a Compact GC 4.0 (Global Analyser

Solutions) equipped with two Rt-UBond columns (2 m × 0.32 mm and 8 m × 0.32 mm) and a flame ionization detector (FID).

The experiment involved a calibration, a propane dehydrogenation (PDH), and an oxidative regeneration phase. The Eu^{3+}-based thermometer was calibrated in inert atmosphere, under He flow (8 mL min^{-1}). During the calibration the temperature was lowered to 560 °C at a rate of 2°C min^{-1}, to stabilize for 10 min, after which the temperature was increased to 600 °C at a rate of 2°C min^{-1}, to stabilize for 10 min at this temperature. Then, the temperature was lowered to 580 °C at a rate of 2°C min^{-1} and stabilized for 20 min, leading up to a total calibration time of 80 min. For the remaining experimental time, the oven temperature and the total gas flow rates were maintained at 580 °C and 8 mL min^{-1}, respectively. The calibration was followed by the PDH phase where propane (60 min), He (10 min), propane (60 min), and He (10 min) were flowed through the reactor. The regeneration started upon the introduction of a 50% O_2 in He mixture (30 min), after which He was flowed through the reactor (10 min).

The combined catalyst and temperature-sensor particles were monitored with alternating time-gated (or time-integrated) Boltzmann thermometry and reflection spectroscopy. The quartz reactor was equipped with a rectangular window that was aligned with a hole in the oven that allowed for the study of the catalyst and temperature-sensor particles with a reflection probe (Avantes, FCR-7UVIR400-2.5-BX-6X350-HTX). The probe delivered light to the sample via six 400 µm diameter fibers, while the luminescence of the illuminated spot was collected with one 400 µm diameter fiber. The crosscut of the probe fibers (Figure S1) illustrates that the excitation fibers were positioned on the corners of a hexagon, with the collection fiber in the center. The probe effectively illuminated numerous catalyst and temperature-sensor particles with a spot of 5 millimeters. Two ends of a bifurcated fiber-optic cable (Avantes, FCB-UV400-2-SR-SI) were used to combine the light of a 375 nm modulated continuous wave (CW) laser (HÜBNER Photonics, Cobolt 06-01-MLD, Extinction ratio of $>10^7$ to 1) and a halogen white light source (Avantes, Avalight-DHS) into one fiber. The bifurcated fiber was coupled to the reflection probe with a 1000 µm core fiber (Thorlabs, M59L01), to allow for optimal light incoupling to the six excitation fibers of the reflection probe (Figure S1). The laser power at the head of the probe was 1.6 mW (Thorlabs, S401C power meter, PM100D controller). The collected luminescence was delivered to a spectrometer with a 250 µm slit, a 400 nm longpass filter, and a 150 lines per mm grating (Oxford Instruments, Andor Shamrock 303i) that was equipped with an intensified scientific complementary metal-oxide semiconductor (sCMOS) camera (Oxford Instruments, Andor iStar-sCMOS-18U-E3).

Alternating time-gated thermometry and reflection spectroscopy experiments were performed using a complex measurement scheme. The camera controlled the modulation of the laser and the shutter of the white lamp with two transistor-transistor-logic signal (TTL) output channels. The camera triggered the laser to deliver block-pulse excitation of 500 Hz with a 20% duty cycle, to be in the on-state from 0 to 0.4 ms in the 2 ms period. The time gate of the camera was opened to collect the luminescence between 0.5 and 1.9 ms in the period. The signal of 2500 laser pulses was integrated to yield the time-gated luminescence spectrum. The time-integrated spectra of Figure 1 were recorded with a continuously opened time gate. Thereafter, the camera triggered the shutter of the white lamp to be in the on-state for 1s. The time gate of the camera was opened for 1 s to collect the reflected signal. The total measurement time of this sequence added up to 6 s, but software related delay between measurements caused the sequence to take about 7 s. The temperature traces shown in this

work are obtained after averaging the temperature data every 40 s. The software (Oxford Instruments, Solis Software) could not facilitate this measurement scheme, so the camera was controlled with a Python script (available upon request). The catalyst and temperature-sensor material were heated up to 580 °C, after which it resided in a He flow. During this step, background spectra were recorded in the absence of the excitation sources. These background spectra were subtracted from the spectra recorded during the experiments. Hereafter, the calibration phase started. At the same time, the online GC program as well as the alternating time-gated thermometry and reflection spectroscopy measurements were initiated. The experiment was repeated seven times, using gain levels in the 1000–1500 range to collect sufficient signal intensity without saturating the detector. All experiments were conducted with a reactor-probe distance of 0 mm, except for experiment 7, which was performed with a reactor-probe distance of 4 mm.

Reference Measurements

Quartz reactors were filled with white and black reference materials, PTFE and carbon black powder, respectively, to record reference reflection spectra of the white lamp (1 s integration time) with the setup described above. The effect of the positioning of the reflection probe relative to the reactor was assessed by measuring the reflection spectrum of the white lamp on the white reference sample. A reflection spectrum was measured by placing the probe from 0 to 7 mm from the reactor in 1 mm increments. Additionally, the reactor was tilted in 6 equal increments from 0 to 45° at reactor-probe distances of 0 and 4 mm.

The time dynamics of the signal of the catalyst and temperature-sensor particles—in the quartz reactor at 580 °C—upon pulsed laser excitation were probed with two time-gate-scan measurements. In the short experiment, the signal resulting from excitation with a 375 nm block pulse (500 Hz, 2 ms period, 20% duty cycle) was recorded by scanning the center of the time gate (50 µs width) in 39 increments of 50 µs from 75 µs to 1975 µs. The settings were such that the laser was in the on-state from 0 to 0.4 ms in the 2 ms period. At each step, the signal was integrated for 5000 pulses, using a gain level of 2000. In the long experiment, the signal resulting from excitation with a 375 nm block-pulse (50 Hz, 20 ms period, 20% duty cycle) was recorded by scanning the center of the time gate (50 µs width) in 300 increments of 50 µs from 0.075 to 15.025 ms. The settings were such that the laser was in the on-state from 2 to 6 ms in the 20 ms period. At each step, the signal was integrated for 500 pulses, using a gain level of 2000.

The influence of the duty cycle and the period length of the time-gated Eu^{3+} luminescence count rate of our temperature-sensor and catalyst particles was studied under the same conditions. In all cases, the time gate of the detector opened 100 µs after a laser pulse and closed 100 µs before the next laser pulse, the individual measurements took 5 s and used a gain level of 2000. We measured for periods ranging from 1 to 5 ms in 0.25 ms increments using fixed duty cycles of 20 and 40%. In another measurement sequence, we measured for duty cycles from 10 to 80% using a fixed period duration of 2 ms.

Gas Chromatography Results

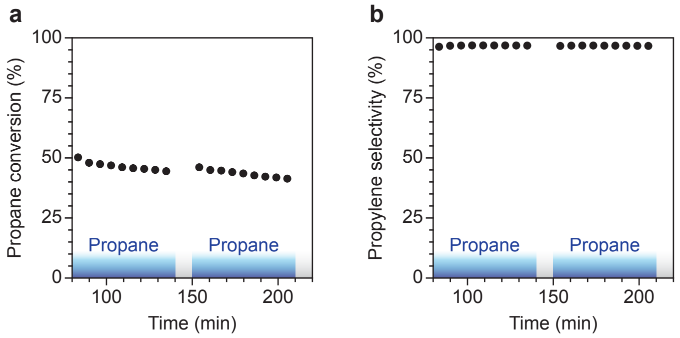

Figure S2. Online gas chromatography (GC) results of the propane dehydrogenation phase with catalyst and temperature-sensor material PtSn/Al_2O_3–Y_2O_3 :Eu^{3+} (150–425 µm) at 580 °C. (a) Propane conversion as function of time. (b) Propylene selectivity as function of time. The formation of methane, ethane, and ethylene was also observed. The GC data was recorded during the experiment, as discussed in Figure 1. The periods under propane and He flow are marked with blue and grey rectangles, respectively.

The online gas chromatography (GC) results—recorded during the experiment discussed in Figure 1—are shown in Figure S2. The conversion of propane decreased from 50% to 40% over the course of the reaction as coke gradually blocked the active sites of the catalyst material (Figure S2a). The selectivity towards the desired product propylene was ~97% over the course of the experiment (Figure S2b). Besides propylene, the products of side- and cracking reactions, methane, ethane, and ethylene were also observed, but they are minor and in the range of 0.2–2.0% selectivity. All other operando spectroscopy experiments in this work were executed in the same fashion, yielding similar online GC results.

Background Correction of the Time-Integrated Spectra

Background fluorescence skewed temperature sensing based on the luminescence intensity ratio (LIR) of the Eu^{3+} phosphor. The Eu^{3+} luminescence spectra recorded during PDH, depicted in Figure 1d, showed that coking gradually reduced the Eu^{3+} luminescence intensity. In the meantime, the background fluorescence intensity increased slightly. As a result, the total emission intensity in the $^5D_1$ spectral region (522–559 nm) increases relatively to the $^5D_0$ spectral region (605–621 nm).

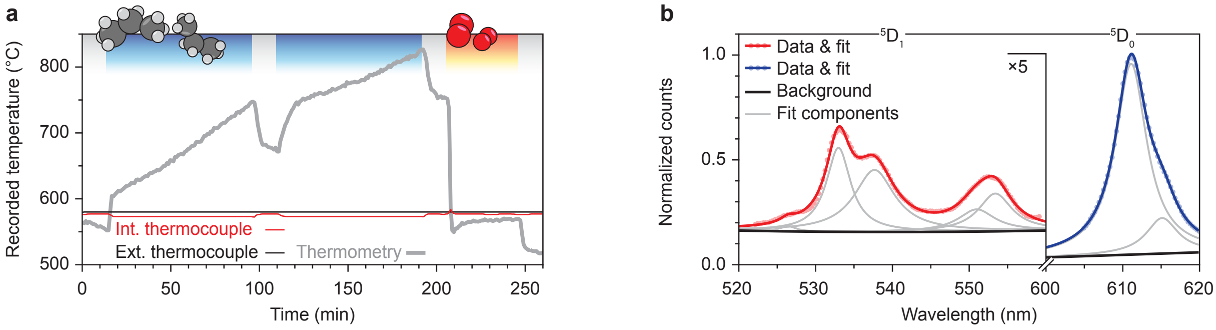

Figure S3. Operando luminescence thermometry for propane dehydrogenation (PDH) catalysis and the problem of background fluorescence. (a) Recorded temperature during the experiment using an external thermocouple (black), an internal thermocouple (red), and luminescence thermometry (grey). The temperature is determined using the luminescence intensity ratio of $^5D_1$ (522–559 nm) and $^5D_0$ emission (605–621 nm) and a calibration analogous to Figure 1 that yielded a ΔE of 716 cm^{-1}. All temperature traces are obtained by averaging the temperature data every 40 s. (b) Spectral fitting method for the $^5D_1$ and $^5D_0$ spectral region. The average spectrum recorded between 25 and 35 min is shown as datapoints. The result of the fitting procedure is shown as the red ($^5D_1$ spectral region) and blue ($^5D_0$ spectral region) solid lines together with the shape of polynomial background (black) and the contribution of individual emission bands (grey).

Figure S3a shows that determining the temperature, only using the luminescence intensity ratio of $^5D_1$ (522–559 nm) and $^5D_0$ emission (605–621 nm) yields enormous temperature readout artifacts. The changing ratio between background fluorescence and Eu^{3+} luminescence during PDH yields an apparent temperature of 825 °C after 210 min, roughly 250 °C higher than the temperature registered with the internal thermocouple.

We use a background correction—based on a spectral fitting method illustrated in Figure S3b—to compensate for the changing background fluorescence. Both spectral regions of interest (522–559 nm and 605–621 nm) of the average calibration spectrum (25–35 min) are modeled as a 2nd-degree polynomial function for the background fluorescence, together with a linear combination of five (522–559 nm) or two (605–621 nm) Lorentzian line shapes,[2] with positions p_i, widths w_i, and areas h_i, for the Eu^{3+} luminescence features:

$$f(x) = ax^2 + bx + c + \sum_i \frac{h_i}{\pi} \frac{w_i/2}{(x-p_i)^2 + (w_i/2)^2}. \qquad \text{Eq. S1}$$

In the next step, the found optimized parameters for the Lorentzian line shapes of the average calibration spectrum are maintained constant. All spectra in the data set are fitted to a 2nd-degree polynomial function for the background and a scaling factor n for the Eu^{3+} Luminescence features:

$$g(x) = ax^2 + bx + c + n\sum_i \frac{h_i}{\pi} \frac{w_i/2}{(x-p_i)^2 + (w_i/2)^2}. \qquad \text{Eq. S2}$$

This function contains the optimizable parameters a, b, c, and n. The luminescence intensity ratio of a spectrum is determined after subtracting the two 2nd-degree polynomial functions from the spectral regions of interest.

Spectral Overlap Between $^5D_0$ and $^5D_1$ Emission and its Influence on the Calibration

In this section, we estimate the effect of spectral overlap between $^5D_0$ and $^5D_1$ emissions on our thermometry experiments. The $^5D_0$ emission peak at 610 nm, which we use in our definition of the LIR, may contain a small contribution from the $^5D_1$ level. Indeed, the $^5D_1 \rightarrow {}^7F_4$ transition has nearly the same energy (16204 cm^{-1}; 617 nm; centers of gravity in LaF_3) as the $^5D_0 \rightarrow {}^7F_2$ transition (16267 cm^{-1}; 615 nm).[3]

We can estimate the intensity of the $^5D_1 \rightarrow {}^7F_4$ emission, which is hidden underneath the dominant signal from the $^5D_0 \rightarrow {}^7F_2$ transition, from a comparison with the clearly resolved $^5D_1 \rightarrow {}^7F_1$ emission intensity at 532 nm. Judd-Ofelt reduced matrix elements are $|U^{(4)}|^2$ = 0.0028 for $^5D_1 \rightarrow {}^7F_4$ and $|U^{(2)}|^2$ = 0.0025 for $^5D_1 \rightarrow {}^7F_1$, while all other matrix elements are 0.[3] The Judd-Ofelt intensity parameters for $Y_2O_3{:}Eu^{3+}$ are $\Omega_2 = 6.31 \times 10^{-20}\ \mathrm{cm}^2$ and $\Omega_4 = 0.66 \times 10^{-20}\ \mathrm{cm}^2$.[4] This leads to an estimated intensity of the $^5D_1 \rightarrow {}^7F_4$ emission band of 10% relative to the $^5D_1 \rightarrow {}^7F_1$ emission intensity. Figure 1e shows that the $^5D_1 \rightarrow {}^7F_1$ emission intensity (at 532 nm) is approximately 10% relative to the $^5D_0 \rightarrow {}^7F_2$ intensity (at 610 nm). Hence, we estimate that the relative contribution of the overlapping $^5D_1 \rightarrow {}^7F_4$ emission at 610 nm is 1% relative to the $^5D_0 \rightarrow {}^7F_2$ intensity. For our further considerations, we multiply this estimate by ×10 to be very conservative and calculate the consequence of spectral overlap in a strongly exaggerated scenario.

We investigate the influence of a 10% overlap between $^5D_1$ and $^5D_0$ on our calibration. With an overlap of $f = 0.1$, the luminescence intensity ratio does not follow Botlzmann behavior exactly, but instead is distorted:

$$\mathrm{LIR}_{\text{with overlap}} = C\,\frac{\mathrm{e}^{-\Delta E/k_\mathrm{B}T}}{(1-f)+f\mathrm{e}^{-\Delta E/k_\mathrm{B}T}}, \qquad \text{Eq. S3}$$

where C is a prefactor containing the energies of the emissions and the degeneracies of the levels, ΔE is the energy difference between the $^5D_1$ and $^5D_0$ levels, and $k_\mathrm{B}T$ is the thermal energy. The term $f\mathrm{e}^{-\Delta E/k_\mathrm{B}T}$ in the denominator describes the contribution of $^5D_1$ emission at 610 nm. We know that $C \approx 3.5$ from our spectral measurements (Figure 1) and $\Delta E = 1740\ \mathrm{cm}^{-1}$.[2,3]

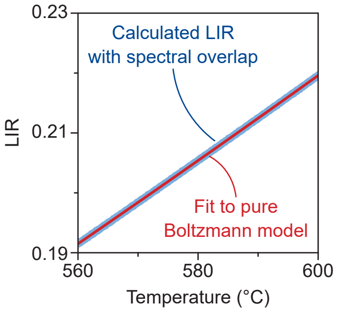

Figure S4. The effect of $^5D_1$ and $^5D_0$ spectral overlap between $^5D_1$ and $^5D_0$ emissions. Blue data points: calculated $\mathrm{LIR}_{\text{with overlap}}$ for $C = 3.5$, $\Delta E = 1740\ \mathrm{cm}^{-1}$, and $f = 0.1$. Red line: fit to a pure Boltzmann model with $C_\mathrm{eff} = 3.8$ and $\Delta E_\mathrm{eff} = 1730\ \mathrm{cm}^{-1}$.

Figure S4 shows the theoretical $\mathrm{LIR}_{\text{with overlap}}$ as a function of temperature between 560°C and 600°C, which is the range where we perform our measurements and our calibration. The red line is a fit to a pure Boltzmann model:

$$\mathrm{LIR}_{\mathrm{Boltzmann}} = C_{\mathrm{eff}}\, \mathrm{e}^{-\Delta E_{\mathrm{eff}}/k_{\mathrm{B}}T}. \quad \text{Eq. S4}$$

We observe that the model fits the data very well. The distortions due to spectral overlap are entirely absorbed into the fitted value of an "effective prefactor" $C_{\mathrm{eff}} = 3.8$ and an "effective energy difference" $\Delta E_{\mathrm{eff}} = 1730\ \mathrm{cm}^{-1}$. The effective energy difference is lower than the real energy difference by as little as $10\ \mathrm{cm}^{-1}$.

By using the fitted curve $\mathrm{LIR}_{\mathrm{Boltzmann}}$ as a calibration, the maximum systematic error in temperature for measurements on the range between 560°C and 600°C is as small as $3 \times 10^{-3}\ \mathrm{K}$. We conclude that our use of a pure Boltzmann model is a satisfactory procedure to account for the influences of spectral overlap.

As a side note: this procedure would continue to work well even if the overlap is very much larger. For example, the maximum systematic temperature error is $0.15\ \mathrm{K}$ for $f = 0.9$, but the effective energy difference becomes unphysical at $\Delta E_{\mathrm{eff}} = 1177\ \mathrm{cm}^{-1}$. Thermometry over a broader temperature range is affected more by spectral overlap.

Optimization of Operando Time-Gated Luminescence Thermometry

Operando time-gated luminescence thermometry, for background fluorescence suppression, benefits from optimization of the Eu^{3+} luminescence count rate. In such an experiment, we can vary the duty cycle of the laser, the period length, and the delay time between excitation and detection. A mathematical description of the excited state population of the Eu^{3+} phosphor, with the duty cycle and period length as input parameters, facilitates the optimization of these parameters.

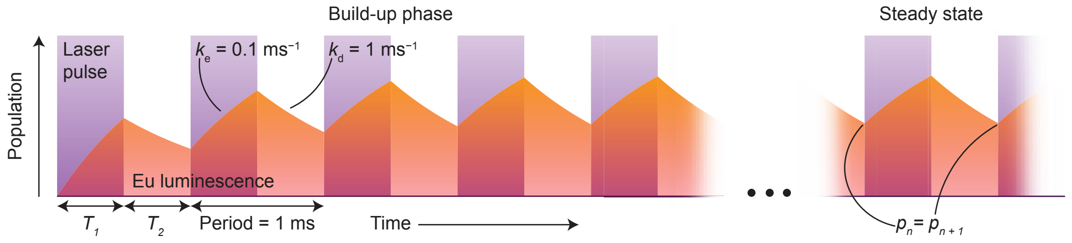

Figure S5. Ingrowth of excited-state Eu^{3+} population upon block-pulse excitation. The excited-state population rises during the block pulse that is on for the length of T_1. The population decays exponentially during the off-period of the laser for a duration T_2. In this realistic case—with a period ($T_1 + T_2$) of 1 ms, a duty cycle of 50%, an excitation rate k_e of 0.1 ms^{-1}, and a decay rate k_d of 1 ms^{-1}—the Eu^{3+} excited state is not depleted fully at moment $t = T_1 + T_2$. As a result, the excited-state population at the beginning of each period grows until reaching a steady state after n periods.

We describe the ingrowth of population $p(t)$, illustrated in Figure S5, of the excited state of a two-level system during a laser block pulse with excitation rate k_e and decay rate k_d with a rate equation:

$$p'(t) = -k_\mathrm{d}\, p(t) + k_\mathrm{e}[1 - p(t)]. \qquad \text{Eq. S5}$$

In the case of incomplete luminescence depletion, a finite excited-state population remains at the beginning of laser pulse n: $p(0) = p_n$. With this boundary condition, the solution of differential equation is:

$$p(t) = p_n \mathrm{e}^{-(k_\mathrm{d}+k_\mathrm{e})t} + \frac{k_\mathrm{e}}{k_\mathrm{d}+k_\mathrm{e}}\left[1 - \mathrm{e}^{-(k_\mathrm{d}+k_\mathrm{e})t}\right] \quad ; \quad 0 < t \leq T_1. \qquad \text{Eq. S6}$$

The population rises according to Eq. S6 until it reaches a population $p(T_1)$ at $t = T_1$, after which the population decays exponentially during the off-state of the laser until $t = T_1 + T_2$. The population at the end of the period, at $t = T_1 + T_2$, is expressed as:

$$p(T_1 + T_2) = p(T_1)\mathrm{e}^{-k_\mathrm{d}T_2} \quad ; \quad T_1 \leq t \leq T_1 + T_2. \qquad \text{Eq. S7}$$

The steady-state population is reached when the population at the beginning of a period is equal to the end of the period, $p_{n+1} = p_n$, after n periods. This condition is met when

$$\left\{p_n \mathrm{e}^{-(k_\mathrm{d}+k_\mathrm{e})T_1} + \frac{k_\mathrm{e}}{k_\mathrm{d}+k_\mathrm{e}}\left[1 - \mathrm{e}^{-(k_\mathrm{d}+k_\mathrm{e})T_1}\right]\right\}\mathrm{e}^{-k_\mathrm{d}T_2} = p_n, \qquad \text{Eq. S8}$$

which has the solution:

$$p_n = \frac{k_\mathrm{e}}{k_\mathrm{d}+k_\mathrm{e}} \frac{1-\mathrm{e}^{-(k_\mathrm{d}+k_\mathrm{e})T_1}}{\mathrm{e}^{-k_\mathrm{d}T_2} - \mathrm{e}^{-(k_\mathrm{d}+k_\mathrm{e})T_1}}. \qquad \text{Eq. S9}$$

Filling in Eq. S9 into Eqs. S6 and S7 provides the full expressions of the rise and decay of the excited-state population in the steady-state regime. These expressions contain parameters k_e, k_d, T_1, and T_2. The first two parameters can be found experimentally, while the latter two parameters dictate the

period length and the duty cycle of the laser during a time-gated luminescence thermometry experiment.

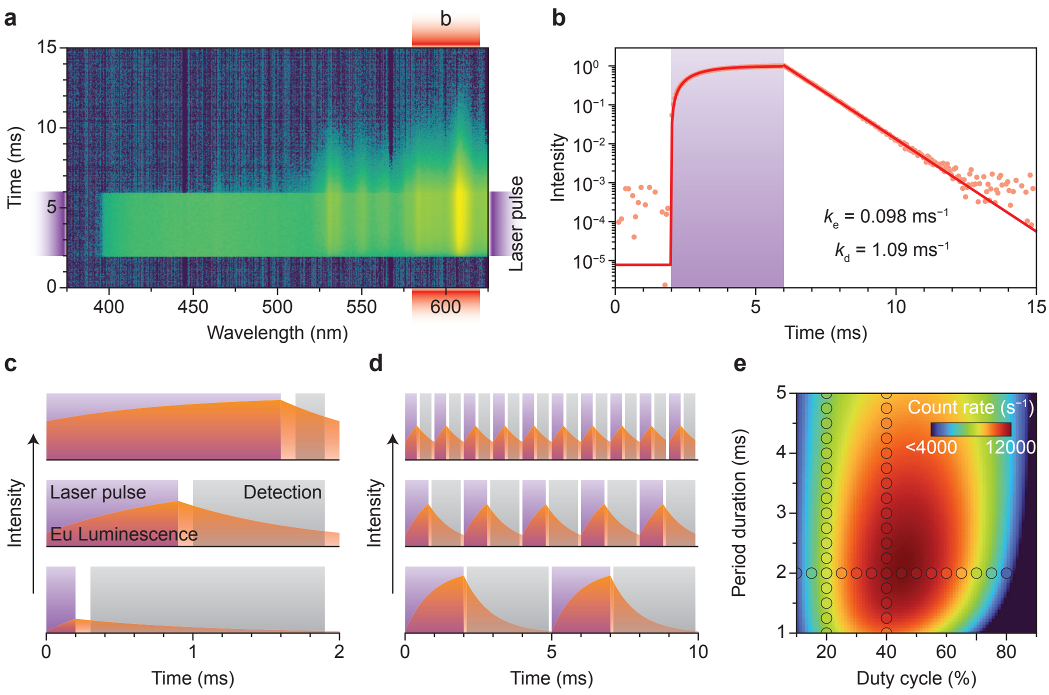

Figure S6. Optimization of time-gated luminescence thermometry for the catalyst and temperature-sensor material PtSn/Al_2O_3–Y_2O_3 :Eu^{3+} (150–425 µm) at 580 °C. (a) Heatmap of time-resolved spectroscopy of the catalyst and temperature-sensor material. The signal resulting from excitation with a 375 nm block-pulse (50 Hz, 20 ms period, 20% duty cycle) is recorded by scanning the center of the time gate (50 µs width) in 300 increments of 50 µs from 0.075 to 15.025 ms. The settings were such that the laser was in the on-state from 2 to 6 ms in the 20 ms period. The intensity is plotted on a logarithmic scale (b) Normalized time trace of the Eu^{3+} luminescence (580–620 nm) from panel a (red points). The time trace is shown together with the results of the fit of the excitation (0–6 ms, Eq. S11) and decay (6–15 ms, Eq. S10) phases of the experiment (red line). The laser pulse is illustrated with the purple rectangle. (c) Theoretical effect of duty cycle on the time-gated luminescence count rate with a fixed period length of 2 ms. (d) Theoretical effect of period length on the time-gated luminescence count rate with a fixed duty cycle of 40%. In panels c and d, the laser pulse is illustrated with purple rectangles, the Eu^{3+} luminescence with the orange traces, and the on-time of the detector with grey rectangles. The dynamics of the Eu^{3+} luminescence are based on Eqs. S6 and S7, using the observed k_e and k_d. The time gate of the detector opens 100 µs after a laser pulse and closes 100 µs before the next laser pulse to fully reject background fluorescence. (e) Theoretical heatmap of the time-gated count rate as a function of duty cycle and period duration. The calculation is made using the same parameters as in panels c and d. The colored circles, with black outlines, show the experimental results of count rate measurements using a range of settings, on the same color scale. The theoretical heatmap is scaled such that its maximum equals the count rate of the 2 ms period and 40% duty cycle measurement. The experimental count rates are determined using the average intensities (525–605 nm) of the time-gated luminescence spectra.

Figure S6 illustrates the optimization of time-gated luminescence thermometry for the catalyst and temperature-sensor material particles under study. The time-dynamics of Eu^{3+} luminescence upon block-pulse excitation are shown in Figure S6a. The Eu^{3+} luminescence intensity rises during the on-time of the laser and decays exponentially during the off-time (Figure S6b). The time trace of the decay (580–620 nm, 6–15 ms) is described by exponential decay with a scaling factor I_0 for the luminescence intensity at the point where the laser switches of, with some constant background intensity B:

$$f(t) = I_0 e^{-k_d t} + B. \qquad \text{Eq. S10}$$

Fitting this function to the time trace (6–15 ms) yields a k_d of 1.09 ms^{-1}. Plugging $p_n = 0$ in Eq. S6, adding a scaling factor I_1, and some constant background intensity B yields an expression for the rise:

$$g(t) = I_1\left[1 - e^{-(k_d+k_e)t}\right] + B. \qquad \text{Eq. S11}$$

Fitting this function to the rise part of the time trace (2–6 ms) yields a k_e of 0.098 ms^{-1}. Plugging the found k_e and k_d into Eqs. S6 and S7 yields a theoretical description of the Eu^{3+} luminescence dynamics of our sample leaving the duty cycle and period length as optimizable parameters.

We use the Eu^{3+} and organic background fluorescence dynamics to determine the time gating that yields an optimal Eu^{3+} luminescence count rate. Figure 2d showed that switching on the detector 100 µs after and 100 µs before a laser pulse is sufficient to record background-fluorescence-free spectra. Figure S6c illustrates that a laser period of 2 ms and duty cycle of 40% yield an optimal time-gated Eu^{3+} luminescence count rate. A shorter duty cycle leads to insufficient build-up of excited-state population and lower signal intensity during the active time of the detector, while a longer duty cycle leaves too little on time for the detector to collect Eu^{3+} luminescence signal. Figure S6d illustrates that a longer laser period leads to significant decay of Eu^{3+} luminescence signal during the on time of the detector, which lowers the overall count rate. A shorter period effectively shortens the fraction of the time during which the detector can be switched on, due to the fixed 100 µs interval between excitation and detection. As a result, the count rate drops with shorter periods.

The theoretical time-gated count rates for relevant combinations of duty cycles and period durations are mapped out and compared to experimental data in Figure S6e. The experimental data (colored circles, with black outlines) agrees with the theoretical model, proving its effectivity for selecting the proper measurement settings. Using a duty cycle of 40% in combination with a period duration of 2 ms yields the optimal count rate. In this work, we used a 20% duty cycle in combination with a 2 ms period for the operando time-gated luminescence thermometry experiments. Lowering the duty cycle from 40 to 20% decreases the count rate by only 27%, while using only half the irradiance, decreasing the probability of sample heating due to the laser.

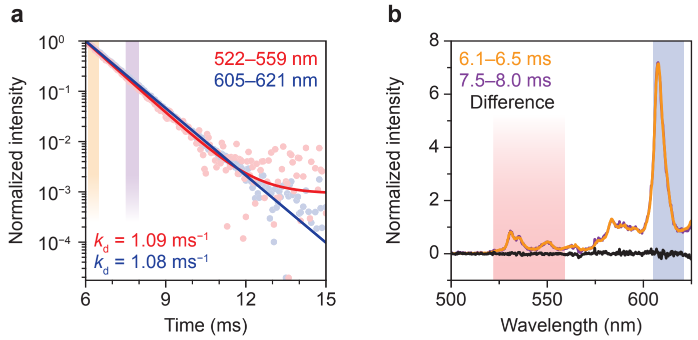

Figure S7. The effect of time gating on the Eu^{3+} luminescence spectrum. This figure uses the time-resolved spectroscopy dataset presented in Figure S6. (a) Normalized time traces of the Eu^{3+} luminescence recorded between 522–559 nm ($^5D_1$ emission; red) and 605–621 nm ($^5D_0$ emission; blue). The time traces are shown together with the results of the fit of the decay (Eq. S10). (b) Normalized Eu^{3+} luminescence spectra recorded between 6.1–6.5 ms (orange) and 7.5–8.0 ms (purple). The spectra are normalized to a constant area. The difference between the two spectra is shown illustrated with the black curve. The colored rectangles in both panels correspond to the wavelength and time boundaries.

In general, time gating eliminates short-lived background fluorescence to produce clean spectra of longer-lived luminescence. However, it is good to note that time gating would skew the combined luminescence spectrum of two emitters with different luminescence lifetimes, resulting in distorted intensity ratios of their spectral features. Conveniently, our Boltzmann thermometer is not sensitive to spectral distortions due to time gating, because it emits from two thermally coupled energy levels

($^5D_0$ and $^5D_1$) that are in thermal equilibrium in the temperature regime of our experiments.[5] Hence, the luminescence decay rates of the $^5D_0$ and $^5D_1$ levels should be equal. Indeed, a closer inspection of the time-resolved spectroscopy dataset of the catalyst and temperature sensor material reveals that the luminescence decay rates from $^5D_1$ and $^5D_0$ emission are similar with a k_d of 1.09 and 1.08 ms^{-1}, respectively (522–559 nm and 605–621 nm, Figure S7a). In addition, the shape of the Eu^{3+} luminescence spectrum does not change when the delay time between excitation and acquisition is varied (Figure S7b). For non-Boltzmann thermometers, the lifetimes of emitting levels is typically not the same. Time gating would still be possible, as long as the calibration and the experiment are done with the same time-gating settings.

Correlation Between Catalyst Activity and Reflection

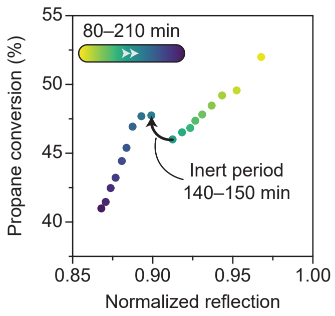

Figure S8. Correlation between catalyst activity and reflection. Averaged and normalized reflection intensity (500–630 nm, Figure 3) plotted against the propane conversion determined with online gas chromatography during the experiment, as discussed in Figure 3.

The buildup of carbon deposits shows in both the online gas chromatography (GC) and the reflection spectroscopy data. The activity of the catalyst—in terms of propane conversion—drops over the course of the first hour of propane dehydrogenation (PDH), is partially restored during the inert period (140–150 min), and decreases again for the second hour of PDH (Figure S2a). The regained activity, during the inert period, might be due to the partial removal of deactivating species from the surface of the catalyst. The average reflected intensity of the white lamp off the surface of the reactor bed (500–630 nm) decreases over the course of the PDH phase due to the darkening of the catalyst material trough coking (Figure 3a). The decreasing reflection correlates strongly with the decreasing activity of the catalyst (Figure S8). Hence, the reflection spectroscopy data provides insights in the condition of the catalyst at work.

Reflectance Correction

We set out to correct the Eu^{3+} luminescence spectra for the changing color (darkening) of the sample using reflection spectroscopy data, recorded during the same experiment. We start from the work by Waters, who adapted the Kubelka–Munk theory to provide an expression for the decrease of the recorded Raman scattering intensity due to absorption and (elastic) scattering by a sample.[6]

Waters uses the approximation that a sample's optical properties are the same at the excitation wavelength and any Raman scattering wavelengths, because Raman scattering typically covers a narrow spectral range. The optical properties were fundamentally described in terms of an absorption coefficient k and a scattering coefficient s. These coefficients determine the reflectance R_∞ of a thick piece of sample and the attenuation of Raman signal. The Raman signal intensity can thus be corrected for sample absorption and scattering based on a measurement of R_∞. This approach proved useful for quantitative Raman spectroscopy of a coking catalyst material.[7]

The diffuse-reflectance-Raman-scattering-intensity relation of Waters needs to be adjusted to make it applicable for our case of luminescence-intensity-ratio based thermometry. The assumption that R_∞—or, more fundamentally, the sample absorption and scattering—is equal for the excitation line (375 nm) and any given luminescence line (500–630 nm) is no longer justified because they are spectrally far apart. Moreover, as we are interested in distortions of the ratio of spectral intensities, we have to account explicitly for differences in the reflectance at different emission wavelengths. We will label the absorption coefficient, scattering coefficient, and reflectance at different wavelengths with subscripts: 0 for the excitation wavelength, and 1 and 2 for two different emission wavelengths.

Waters uses a 1D model for light absorption and back-scattering in a powder sample. The conversion of excitation light into Raman scattering is described by a coefficient ρ. In our case, the conversion is due to luminescence, and can be described by a similar coefficient. We will label this coefficient ρ_1 for luminescence at wavelength λ_1, or ρ_2 for luminescence at wavelength λ_2. Following the derivation of Waters, but explicitly accounting for different optical properties at the excitation wavelength and the luminescence wavelength, Eq. 14 in the work of Waters for the recorded luminescence intensity ψ_1 becomes:

$$\psi_1 = \rho_1 I_0 \frac{(1+R_0)(1+R_1)}{\sigma_0+\sigma_1}; \ \sigma_0 = \sqrt{k_0(k_0+2s_0)}, \sigma_1 = \sqrt{k_1(k_1+2s_1)}. \qquad \text{Eq. S12}$$

Here, k_0 and s_0 are the absorption and scattering coefficients at the excitation wavelength, k_1 and s_1 are the absorption and scattering coefficients at the luminescence wavelength, R_0 is the reflectance at the excitation wavelength, R_1 is the reflectance at the emission wavelength, and I_0 is the excitation intensity.

In our experiments, we want to convert the ratio ρ_1/ρ_2 of luminescence generation coefficients at two different wavelengths, corresponding to emissions from the $^5D_1$ and $^5D_0$ states, to a temperature. The observed luminescence intensity ratio ψ_1/ψ_2 relates to the luminescence generation coefficients as:

$$\frac{\psi_1}{\psi_2} = \frac{\rho_1}{\rho_2}\frac{(1+R_1)}{(1+R_2)}\frac{\sigma_0+\sigma_2}{\sigma_0+\sigma_1}. \qquad \text{Eq. S13}$$

To extract ρ_1/ρ_2 from the recorded ψ_1/ψ_2 and reference measurements of the sample reflectance, we make the reasonable assumption that scattering and absorption at the excitation wavelength are considerably stronger than at the luminescence wavelength ($\sigma_0 \gg \sigma_1, \sigma_2$). In that scenario,

$$\frac{\rho_1}{\rho_2} = \frac{\psi_1}{\psi_2}\frac{(1+R_2)}{(1+R_1)}. \quad \text{Eq. S14}$$

The effective correction procedure uses the observed Eu^{3+} luminescence spectrum $\psi(\lambda)$ and the reflectance spectrum $R(\lambda)$ to construct a reflectance-corrected Eu^{3+} luminescence spectrum $\rho'(\lambda)$:

$$\rho'(\lambda) = \frac{\psi(\lambda)}{1+R(\lambda)}. \quad \text{Eq. S15}$$

This form of the Eu^{3+} luminescence spectrum has the correct shape of the luminescence generation as a function of wavelength, in the limit that $\sigma_0 \gg \sigma(\lambda)$, where $\sigma(\lambda)$ is the attenuation coefficient at emission wavelength λ. Hence, the spectrum $\rho'(\lambda)$ is suitable to calculate luminescence intensity ratios. However, it does not correct the recorded spectrum for changes in the absolute intensities due to sample absorption and scattering at the excitation wavelength, because a factor $(1 + R_0)/\sigma_0$ is missing.

In practice, the reflection spectra recorded during the thermometry experiments are converted to reflectance spectra after considering the effect of light reflection internally in the metal-clad fiber probe and on the quartz window of the reactor. The total recorded reflection intensity is the sum of reflection of the powder sample (constituting catalyst and temperature-sensor material) and the reflection elsewhere in the setup.

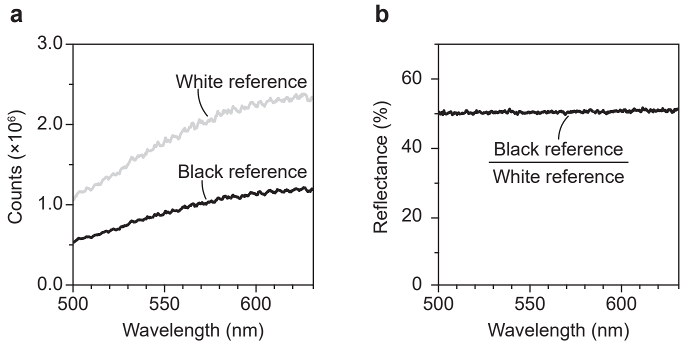

Figure S9. Quantification of the contribution of light reflection on other interfaces than the powder sample. (a) Recorded reflection spectra of the black (carbon black) and white (polytetrafluoroethylene) reference materials in a quartz reactor. (b) Reflectance spectrum of the black reference material. The reflectance spectrum is obtained by dividing the black reference spectrum over the white reference spectrum.

Figure S9 quantifies the contribution of light reflected of the quartz reactor windows. The reflection spectra of black and white reference powders placed in quartz reactors are depicted in Figure S9a. Interestingly, a considerable amount of signal originates from the black reference. Assuming that the black material absorbs all the light, ~50% of the recorded reflection intensity is due to the reflection from elsewhere in the optical setup (Figure S9b). We hypothesize that this could be due to reflections on the quartz reactor walls or internally in the metal rod that holds the fiber probe close to the reactor.

The compensation for the light reflection on the glass and inside the fiber probe modulates the correction of Eu^{3+} luminescence spectra. We extract the reflectance spectra $R(\lambda)$ of the catalyst powder from the recorded reflection spectrum $y_{\mathrm{cat}}(\lambda)$ of the reactor containing catalyst using:

$$R(\lambda) = \frac{R\prime(\lambda) - x}{1 - x}; x = \frac{y_{\text{glass}}}{y_{\text{white}}}; R'(\lambda) = \frac{y_{\text{cat}}}{y_{\text{white}}}. \quad \text{Eq. S16}$$

Here, y_{glass} is the glass reflection spectrum (accounting for all the reflection elsewhere in the setup), y_{white} is the white reference reflection spectrum, and y_{cat} is the reflection spectrum of the catalyst and temperature-sensor material. This reflectance spectrum, including the correction term x, is plugged into Eq. S15.

In practice, the $R'(\lambda)$ spectrum is determined by averaging sets of two sequentially recorded reflection spectra in the operando reflection spectroscopy dataset. Effectively, each Eu^{3+} luminescence spectrum is corrected to yield $\rho'(\lambda)$ using the reflection spectra recorded right before and right after its acquisition. The luminescence intensity ratio of the $^5D_1$ (522–559 nm) to $^5D_0$ (605–621 nm) spectral regions in $\rho'(\lambda)$ are then used to determine the temperature. We assume that x depends on the wavelength, as confirmed by Fig. S5b. Therefore, we use two fit parameters, x_0 and x_1, and optimize their values with a fit procedure (to compute $\rho'(\lambda)$ in the two spectral regions of interest). These two parameters account for the fraction of light reflected off the reactor or in the fiber probe in the spectral regions of the $^5D_0$ and $^5D_1$ emission lines, respectively. The values of x_0 and x_1 are optimized with a fit procedure by minimizing the temperature differences between the inert periods and the after the regeneration (70–80 min, 142–150 min, 213.5–220.5 min, and 228–238 min, Figure 3c). For Figure 3, this yielded $x_0 = 0.474$ and $x_1 = 0.494$, in reasonable agreement with the measurements in Figure S9. The values of x_0 and x_1 are difficult to establish without such an optimization, because the intensities and the shapes of reference spectra strongly depend on the position of the probe relative to the reactor (Figure S10).

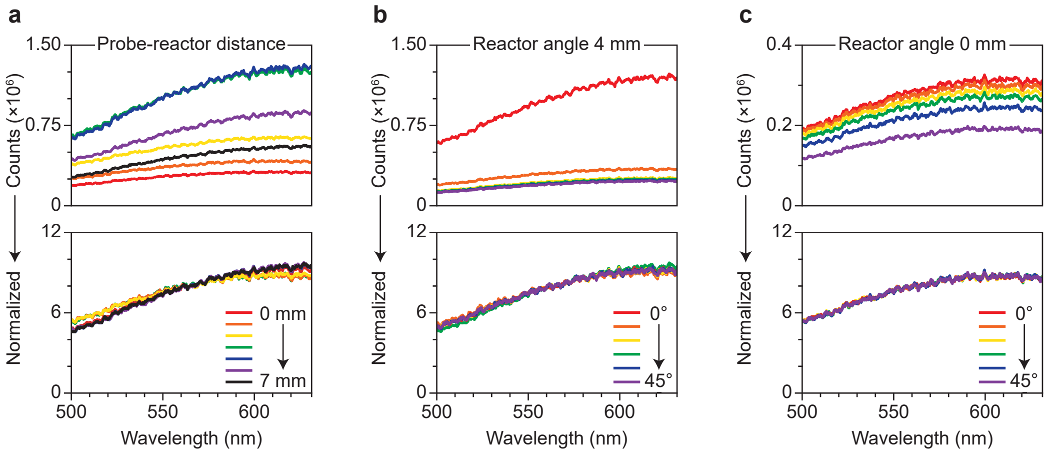

Figure S10. Probe positioning relative to the reactor and the effect on the white reference reflection spectrum. (a) Effect of the probe–reactor distance. A reflection spectrum was measured by placing the probe from 0 to 7 mm from the reactor in 1 mm increments. (b) Effect of the reactor angle at a probe–reactor distance of 4 mm. (c) Effect of the reactor angle at a probe-reactor distance of 0 mm. At both probe-reactor distances the reactor was tilted in 6 equal increments from 0 to 45°. In the tilted series with the probe–reactor distance of 0 mm, a fraction of the probe head was in contact with the reactor. All spectra are normalized such that the integral equals 100.

Figure S10 illustrates that the intensities and the shapes of white reference spectra strongly depend on the position of the probe relative to the reactor. The intensity of the reflection spectrum increases ~5-fold when moving the probe from 0 to 4 mm from the reactor, after which it decreases again (Figure S10a). Normalizing these spectra illustrates that the spectral shape is influenced by the reactor-probe distance too.

The angle at which the reactor is placed also influences the shape and intensity of the white reflectance spectrum, as shown in Figures S10b and S10c. The reflected intensity decreases strongly when tilting the reactor window relative to the probe. This effect is stronger at a reactor–probe distance of 4 mm, compared to the reactor–probe distance of 0 mm.

Precise and reproducible placement of the reactors in the oven (Figure S1) is difficult and complicates the reflectance correction. As a result, positions of the white reference reactor and the reactor used for the thermometry experiments varied from measurement to measurement. These small variations in probe-reactor distances and placement angels strongly influence the reflection spectra. The optimization of x_0 and x_1 for each experiment made the reflectance correction method robust. In future experiments, the setup alignment might be stabilized further with more rigid holders for the probe and the reactor, to ensure that they have a more constant position with respect to each other from measurement to measurement.

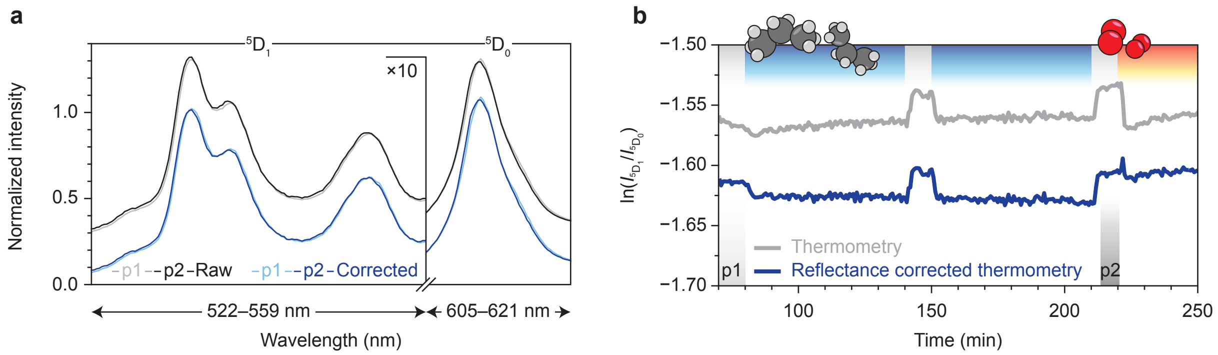

Figure S11 Spectral distortions compensated by the reflectance correction. This figure uses the same data as Figures 2 and 3. (a) Averaged and normalized time-gated Eu^{3+} luminescence spectra recorded during inert periods between 80–70 min (p1) and 213.5–220.5 min (p2). The reflectance-corrected spectra are presented in blue, the raw (uncorrected) spectra are presented with an offset in black. The spectra are normalized to a constant area. (b) Luminescence intensity ratios recorded during the experiment without (grey) and with the reflectance correction (blue). The natural logarithm of the ratio of $^5D_1$ to $^5D_0$ emission (522–559 and 605–621 nm, respectively) is plotted against time. All LIR traces are obtained by averaging the data every 40 s.

The reflectance correction has a subtle effect on the shape of the Eu^{3+} luminescence spectra (Figure S11). The effect of the reflectance correction can be illustrated with Eu^{3+} luminescence spectra recorded during inert periods in He, here the temperature is constant and so should the resulting luminescence intensity ratio (LIR) be as well. However, comparing the averaged Eu^{3+} luminescence spectra of the clean and coked sample (80–70 min and 213.5–220.5 min respectively) reveals that the ratio of $^5D_1$ to $^5D_0$ emission is skewed slightly after coking, resulting in an increased LIR. We attribute this skewed LIR to the color change of the sample. The reflectance correction was designed to mitigate this effect. Indeed, the reflectance-corrected spectra of the clean and coked sample have the similar shapes and LIRs.

Reproducibility and precision of the Method

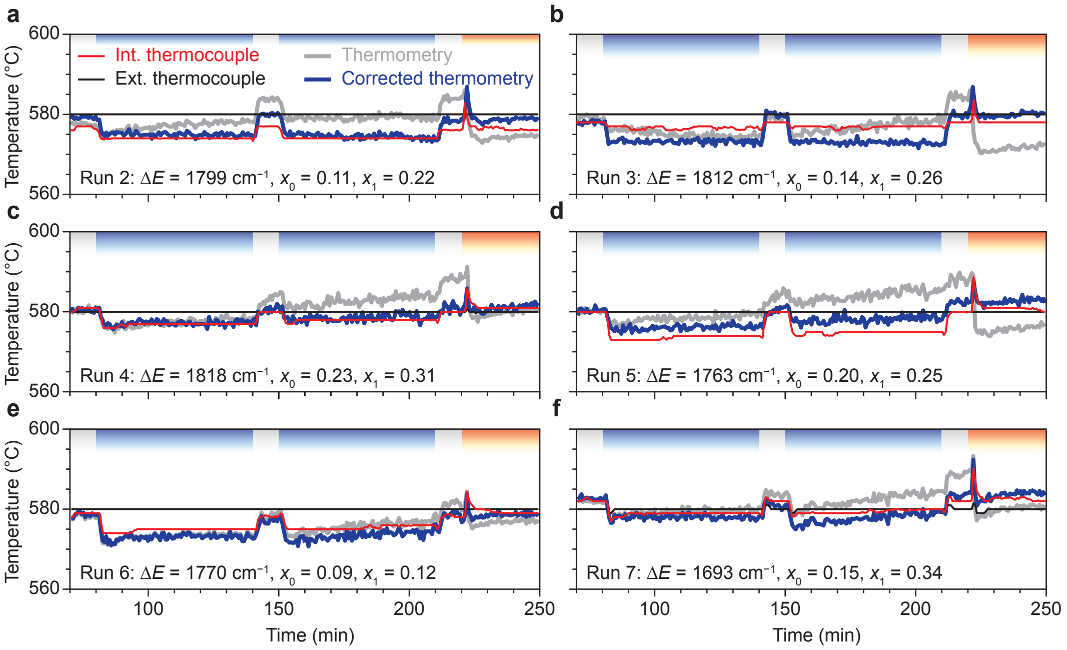

Figure S12. Reproducibility of operando reflectance-corrected time-gated luminescence thermometry for propane dehydrogenation (PDH) catalysis. Recorded temperature during the experiment using an external thermocouple (black), and an internal thermocouple (red), time-gated luminescence thermometry (grey line), and reflectance-corrected time-gated luminescence thermometry (blue line). For each reflectance correction, the values of x_0 and x_1 are optimized with a fit procedure by minimizing the temperature differences between the inert periods and the period after the regeneration (70–80 min, 142–150 min, 213.5–220.5 min, and 228–238 min). All temperature traces are obtained by averaging the temperature data every 40 s.

The reproducibility of the operando reflectance-corrected time-gated luminescence thermometry method is illustrated in Figure S12. In all cases, the uncorrected time-gated thermometry data deviated considerably from the temperature recorded by the internal thermocouple. The correction for the color changes of the sample was successful for all experiments presented in Figure S12. We observe that the corrected temperature is more stable during the inert periods at 70–80 min, 140–150 min, and 210–220 min, compared to the uncorrected temperature. During PDH, the temperature is lower by 5–10 °C. Most notably, the correction procedure brings about the exothermic temperature spike at the start of the regeneration phase, at 220 min, which was obscured by the changing color of the sample. The optimization of x_0 and x_1 from experiment to experiment, to account for the sensitivity to setup alignment, made the thermometry method robust and reproducible. The differences in the magnitude of the endothermic and exothermic effects and the values of x_0 and x_1 from measurement to measurement might be due to minor differences in probe alignment.

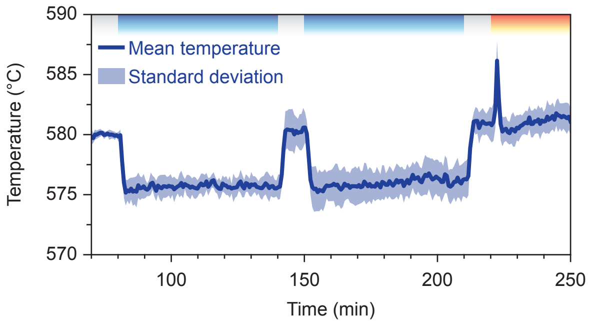

Figure S13. Precision of operando reflectance-corrected time-gated luminescence thermometry for propane dehydrogenation (PDH) catalysis. The temperature trace (blue) is the mean of the offset-corrected temperature traces obtained with the seven reflectance-corrected time-gated luminescence thermometry experiments presented in Figure 3 and Figure S12. Before calculating the mean, each temperature trace is corrected for the varying offset by setting the average temperature recorded between 70 and 80 min to 580 °C. The width of the light-blue shaded area illustrates the standard deviation in the temperature over the seven experiments: $\sigma_t = \sqrt{\langle (T_{tX} - \langle T_{tX} \rangle_X)^2 \rangle_X}$ where T_{tX} is the calculated temperature at time t during experiment X and $\langle \cdot \rangle_X$ denotes averaging over the seven experiments. The average standard deviation on the mean measurement is 1.10 °C.

The systematic errors of luminescence thermometry due to dynamic changes in the optical properties of the sample were mitigated with time gating and the reflectance correction. The luminescence thermometry data accurately tracks the temperature of the sample (Figure S12).

An analysis of the precision of our thermometry method is presented in Figure S13. This analysis is already corrected for a systematic error in the measurements due to deviations of the temperature reading of the internal thermocouple from the oven temperature of 580 °C at the beginning of the experiment. This offset variation is clearly visible when comparing the average luminescence thermometry temperature between 70 and 80 min in Figure S12b to Figure S12f. The luminescence thermometer is calibrated with the temperature readout of the internal thermocouple. The placement of the thermocouple varies from measurement to measurement, resulting in the offset variations. We compensate for the offset variations in our precision analysis. Otherwise, the most dominant difference from measurement to measurement would be the offset variation.

To estimate random errors due to random noise and systematic errors unresolved by our correction procedure, we study the inert periods (70–80 min, 142–150 min, 213.5–220.5 min, 228–238 min) of seven nominally identical experiments (Figure 3 and Figure S12). As inert He flows over the catalyst during these periods, we know the temperature should be constant. We estimate the total error on the measurements from the standard deviation in temperature evaluated over the corresponding inert periods of the seven experiments combined:

$$\sigma_{\mathrm{total}} = \sqrt{\langle \left(T_{tX} - \langle T_{tX} \rangle_{t,X} \right)^2 \rangle_{t,X}}. \qquad \text{Eq. S17}$$

In this notation, T_{tX} is the temperature recorded at time t during experiment X, and $\langle \cdot \rangle_{t,X}$ denotes averaging over all times t within an inert period and all experiments X. We find that σ_{total} ranges from 0.38 °C during the inert period of 70–80 min to 1.59 °C for 142–150 min, with a mean of $\langle \sigma_{\mathrm{total}} \rangle = 1.07$ °C over the four inert periods. σ_{total} is higher when the catalyst is darker (142–150 min and 213.5–

220.5 min), as the spectral data is noisier. The remaining systematic error after our absorption-correction procedure is estimated from

$$\sigma_{\text{syst}} = \sqrt{\langle \left(\langle T_{tX} \rangle_t - \langle T_{tX} \rangle_{t,X} \right)^2 \rangle_X}. \qquad \text{Eq. S18}$$

Here, $\langle\cdot\rangle_t$ and $\langle\cdot\rangle_X$ denote averaging separately over t within an inert period and over the seven experiments X, respectively. Eq. S18 is the variation between measurement outcomes of nominally identical experiments, which have, however, slight differences in alignment. We find that σ_{syst} ranges from 0.02 °C during the inert period of 70–80 min to 1.40 °C for 142–150 min, with a mean of $\langle\sigma_{\text{syst}}\rangle =$ 0.87 °C over the four inert periods.

Even after our correction procedure, the systematic errors are larger than the random errors

$$\sigma_{\text{random}} = \langle \sqrt{\langle (T_{tX} - \langle T_{tX} \rangle_t)^2 \rangle_t} \rangle_X \qquad \text{Eq. S19}$$

due to counting noise, which amount for 0.37 °C for 70–80 min and 0.73 °C for 142–150 min. This highlights the importance for the luminescence community to address systematic errors as a more impactful influence on experiments than random noise.

References

(1) Vogel, R.; Prins, P. T.; Rabouw, F. T.; Weckhuysen, B. M. Operando Time-Gated Raman Spectroscopy of Solid Catalysts. *Catal. Sci. Technol.* **2023**, *13* (22), 6366–6376.

(2) Chang, N. C.; Gruber, J. B. Spectra and Energy Levels of Eu^{3+} in Y_2O_3. *J. Chem. Phys.* **1964**, *41* (10), 3227–3234.

(3) Carnall, W. T.; Crosswhite, H.; Crosswhite, H. M. *National Laboratory Report*; 1977.

(4) Ćirić, A.; Stojadinović, S.; Dramićanin, M. D. Temperature and Concentration Dependent Judd-Ofelt Analysis of Y_2O_3:Eu^{3+} and YVO_4:Eu^{3+}. *Phys. B Condens. Matter* **2020**, *579*, 411891.

(5) Geitenbeek, R. G.; Salzmann, B. B. V.; Nieuwelink, A.-E.; Meijerink, A.; Weckhuysen, B. M. Chemically and Thermally Stable Lanthanide-Doped Y_2O_3 Nanoparticles for Remote Temperature Sensing in Catalytic Environments. *Chem. Eng. Sci.* **2019**, *198*, 235–240.

(6) Waters, D. N. Raman Spectroscopy of Powders: Effects of Light Absorption and Scattering. *Spectrochim. Acta Part A Mol. Spectrosc.* **1994**, *50* (11), 1833–1840.

(7) Tinnemans, S. J.; Kox, M. H. F.; Nijhuis, T. A.; Visser, T.; Weckhuysen, B. M. Real Time Quantitative Raman Spectroscopy of Supported Metal Oxide Catalysts without the Need of an Internal Standard. *Phys. Chem. Chem. Phys.* **2005**, *7* (1), 211-216.
